# Role of the NF-kB signalling pathway in heterotopic ossification: biological and therapeutic significance

**DOI:** 10.1186/s12964-024-01533-w

**Published:** 2024-03-04

**Authors:** Fangzhou Liu, Yike Zhao, Yiran Pei, Fengyu Lian, Hui Lin

**Affiliations:** 1 Department of Pathophysiology, School of Basic Medical Sciences, Jiangxi Medical College, Nanchang University, Nanchang, Jiangxi 330006 China; 2 Queen Mary School, Jiangxi Medical College, Nanchang University, Nanchang, Jiangxi Province 330006 China

**Keywords:** Heterotopic ossification, NF-κb signalling, Molecular mechanism, Treatment

## Abstract

Heterotopic ossification (HO) is a pathological process in which ectopic bone develops in soft tissues within the skeletal system. Endochondral ossification can be divided into the following types of acquired and inherited ossification: traumatic HO (tHO) and fibrodysplasia ossificans progressiva (FOP). Nuclear transcription factor kappa B (NF-κB) signalling is essential during HO. NF-κB signalling can drive initial inflammation through interactions with the NOD‐like receptor protein 3 (NLRP3) inflammasome, Sirtuin 1 (SIRT1) and AMP-activated protein kinase (AMPK). In the chondrogenesis stage, NF-κB signalling can promote chondrogenesis through interactions with mechanistic target of rapamycin (mTOR), phosphatidylinositol-3-kinase (PI3K)/AKT (protein kinase B, PKB) and other molecules, including R-spondin 2 (Rspo2) and SRY-box 9 (Sox9). NF-κB expression can modulate osteoblast differentiation by upregulating secreted protein acidic and rich in cysteine (SPARC) and interacting with mTOR signalling, bone morphogenetic protein (BMP) signalling or integrin-mediated signalling under stretch stimulation in the final osteogenic stage. In FOP, mutated ACVR1-induced NF-κB signalling exacerbates inflammation in macrophages and can promote chondrogenesis and osteogenesis in mesenchymal stem cells (MSCs) through interactions with smad signalling and mTOR signalling. This review summarizes the molecular mechanism of NF-κB signalling during HO and highlights potential therapeutics for treating HO.

## Heterotopic ossification (HO)

HO refers to the presence of ectopic bones in the soft tissue of extraskeletal regions induced by severe external stimuli [1]. The clinical manifestations range from topical pain, swelling, and stiffness to advanced mature bone formation that eventually restricts joint mobilization and thus worsens quality of life [1]. HO can be divided into 2 types: genetic HO and traumatic HO [2, 3].

### Traumatic HO

Traumatic HO (tHO) is often thought of as HO that is caused by injury and occurs most frequently after significant soft tissue injury [4]. In general, HO requires three events, including inflammation, chondrogenic differentiation in mesenchymal stem cells (MSCs) [5], and the establishment of a suitable microenvironment [6, 7]. According to numerous studies, macrophages also play a significant role in regulating the many facets of HO development [8]. Specifically, the molecular mechanisms underlying HO involve several signalling pathways mediated by BMP, HIF1-α (hypoxia inducible factor 1-α), TGF-β (transforming growth factor-β), and RAR (retinoic acid receptor) [3]. Additionally, an increasing number of studies have shown the involvement of nuclear transcription factor kappa B (NF-κB) in multiple cellular processes, especially those that can facilitate inflammation and subsequent bone formation [8, 9].

### FOP

The most common form of genetic HO is fibrodysplasia ossificans progressiva (FOP), which is an autosomal dominant genetic disease that can occur after infection or trauma [10, 11]. FOP is caused by a mutation in the activin A receptor type I (ACVR1) gene [12–14]. The primary cause of FOP is a recurrent gain-of-function mutation (R206H) in the glycine-serine (GS) activation region of the ACVR1/ALK2 type I BMP receptor kinase [15]. Due to the susceptibility of the genetically predisposed immune system to overactivation, FOP patients can be diagnosed with autoinflammatory diseases [16]. The ACVR1 R206H mutation can cause specific functional immune changes with broad impacts, such as increased diffuse and targeted cytokines in peripheral blood monocytes and macrophages [16].

## NF-κB signalling

**Fig. 1:**
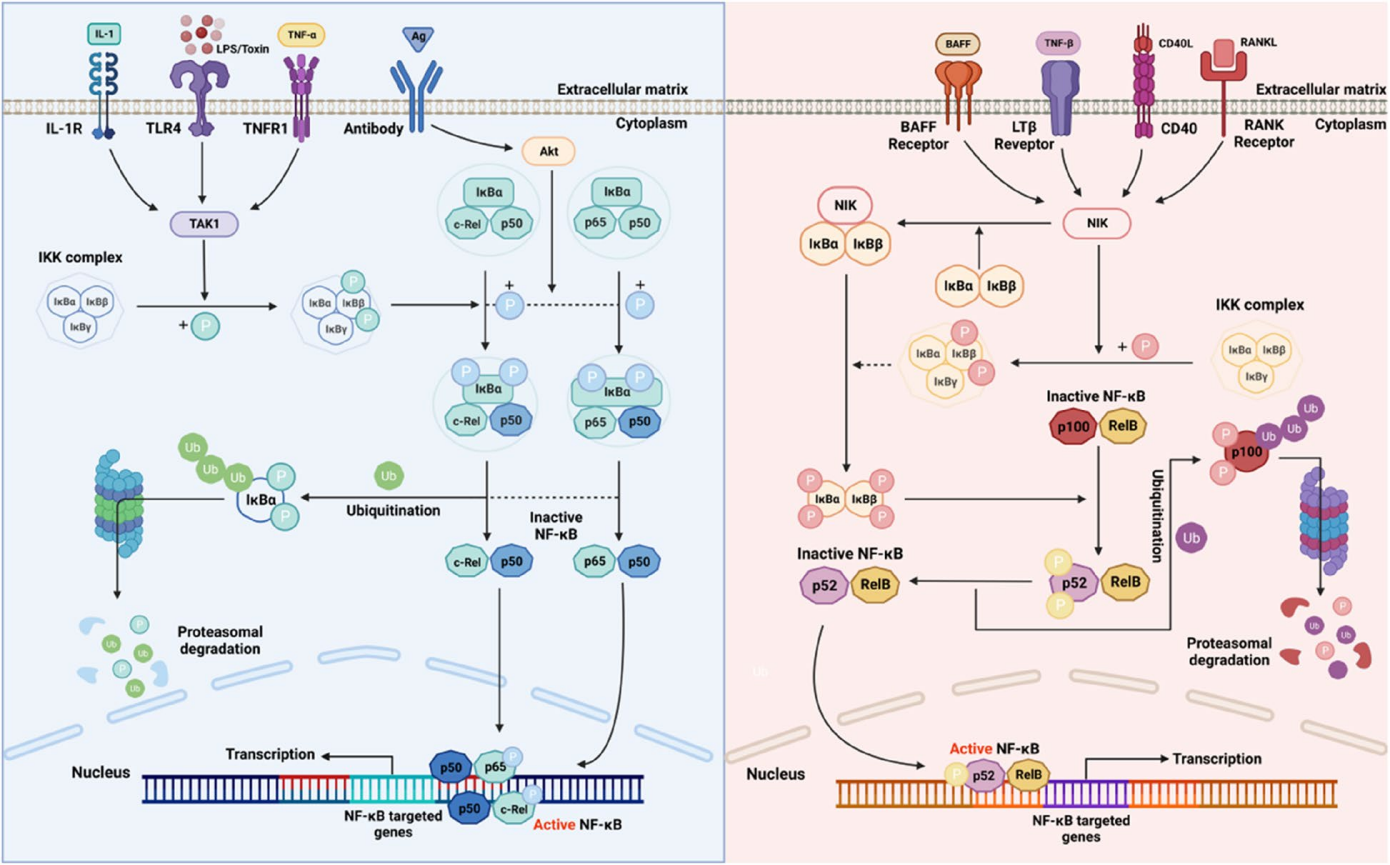
The simplified diagram of the canonical pathway and alternative pathway of the NF-κB signalling pathway (Created with BioRender.com)

NF-κB regulates the differentiation and activation of B cells [17, 18]. There are 5 main factors in the NF-κB family that are expressed in mammals: RelB, c-Rel, p65 (RelA), NF-κB1 (p105/p50), and NF-κB2 (p100/p52). There are two pathways that regulate NF-κB: the traditional pathway, which is regulated by IKKβ and triggered by inflammatory cytokines such as tumour necrosis factor-alpha (TNF-α), lipopolysaccharides (LPS), and interleukin-1β (IL-1β); and the alternative pathway, which is dependent on IKKα and stimulated by CD40, LTβR, and receptor activator of NF-κB, which activate and induce p52/RelB heterodimerization [17–19] (Fig. 1).

## NF-κB signalling in tHO

NF-κB signalling was shown to play an important role in tHO, and it drives inflammation and modulates chondrogenesis and osteogenesis [8]. In this review, we aim to summarize the role of NF-κB signalling in the development of pathological ossification.

### NF-κB signalling in the initial inflammation stage of HO

**Fig. 2:**
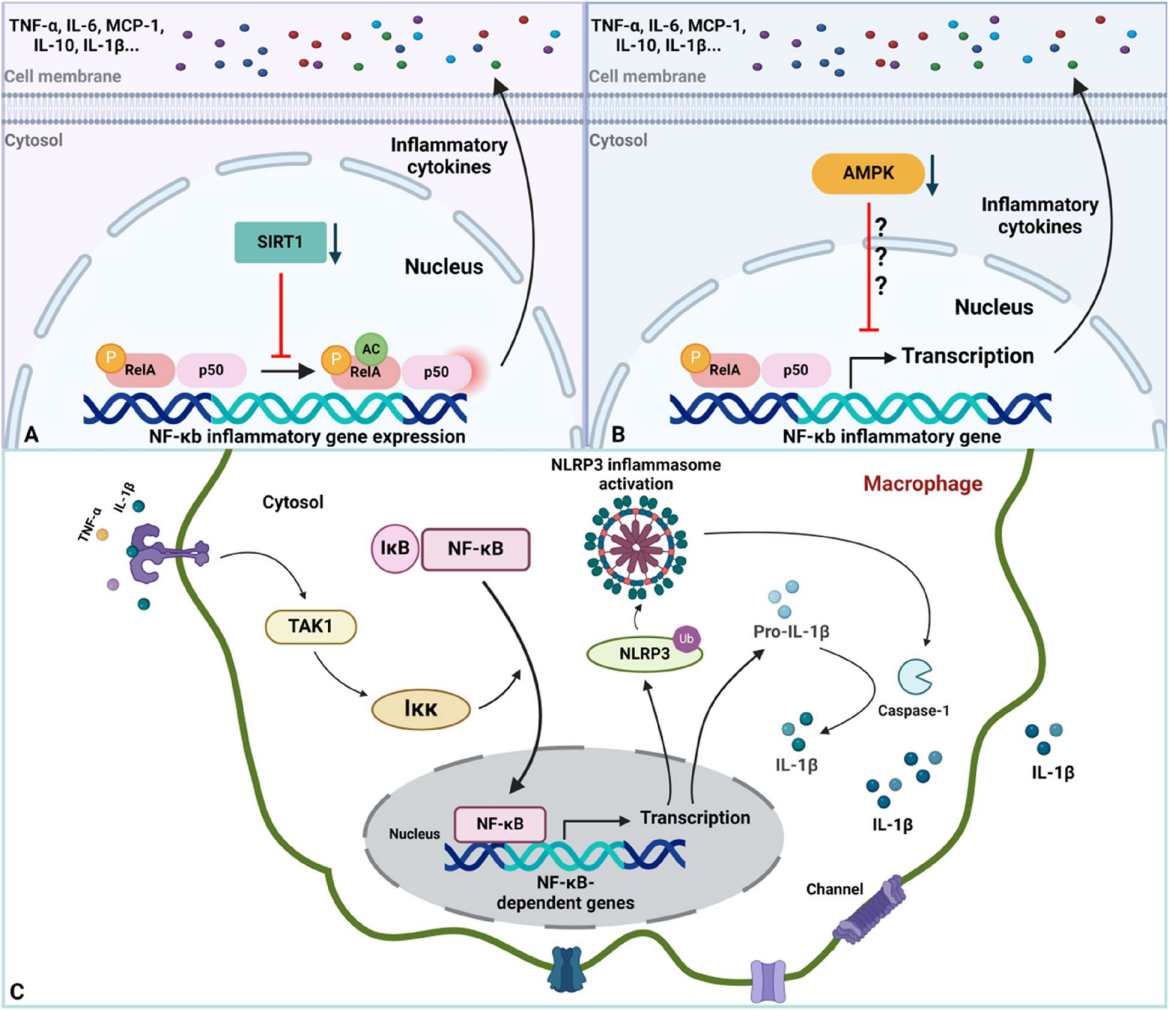
Schematic of NF-κB signalling in macrophages during early inflammation in endochondral ossification. **A** Downregulation of SIRT1 expression disinhibits acetylation of RelA/P65, increasing RelA/P65 activity to promote NF-κB-dependent inflammatory gene expression (TNF-α and IL-6). **B** Downregulation of AMPK expression enhances RelA/P65 activity, enlarging NF-κB-dependent gene transcription (IL-10 and MCP-1). **C** Inactive macrophages can be stimulated to an active state via cytokine-receptor binding, promoting NLRP3 and pro-IL-1β expression. Active NLRP3 inflammasome activates caspase-1 to convert pro-IL-1β into IL-1β, further promoting chondrogenesis (Created with BioRender.com)

Macrophages are considered to be the predominant factors in the innate and adaptive immune responses during the early inflammatory phase of HO, which involves tissue homeostasis remodelling and regulation [20]. Hou et al. discovered that M1 and M2 macrophage infiltration are dramatically increased within injured tissue sites, and their levels peaked on different days after trauma [21]. Not surprisingly, a well-known function of the NF-κB pathway is to modulate inflammatory responses [9]. Generally, NF-κB mediates the expression of many proinflammatory genes in the innate immune system [9, 22]. In general, during the inflammatory stage of HO, sirtuin1 (SIRT1), AMPK and the NLRP3 inflammasome are involved in the NF-κB signalling pathway in macrophages [8, 23–25]. Specifically, the expression of SIRT1 and AMPK, which can inhibit NF-κB signalling, was decreased in macrophages [23–25]. Therefore, this decrease in expression could disinhibit the secretion of proinflammatory cytokines [23–25]. Additionally, macrophage-derived inflammatory cytokines can act in a Toll-like receptor 4 (TLR4)-dependent manner to trigger the activation of NF-κB signalling, thereby promoting NLRP3 inflammasome induction and further proinflammatory cytokine activation and secretion [25] (Fig. 2).

#### SIRT1

SIRT1 is a conserved nicotinamide adenosine dinucleotide (NAD)-dependent protein deacetylase in mammals that performs various biological functions and is predominantly involved in metabolism, ageing and the cell cycle [23, 26–28]. Recently, a growing number of studies have revealed that SIRT1 can coordinate signalling pathways involved in inflammation and the modulation of the immune microenvironment. Furthermore, a study based on animal models with osteolysis examined whether the activation of SIRT1 could alleviate inflammatory responses and block bone resorption, which can provide potential targets for HO treatment [23].

In traumatic HO, SIRT1 was also proven to play immunomodulatory roles, which are associated with NF-κB signalling [23]. There are many SIRT1 substrates, including transcription factors (TFs) for p53, PGC-1 (peroxisome proliferator-activated receptor-γ coactivator) and FOXO (Forkhead box O). Among those, NF-κB-p65 is a known proinflammatory factor that is involved in immune regulation. The acetylation of p65 at the K310 residue is necessary for the complete transcriptional activity of NF-κB and the induction of canonical signalling [23]. As an upstream regulator, SIRT1 can inhibit the acetylation of p65, thereby inhibiting NF-κB-dependent gene expression [29]. Therefore, dysregulation of SIRT1/NF-κB signalling is essential for inflammatory responses in HO, and immunohistochemical staining of HO revealed increased secretion of inflammatory cytokines, including TNF-a, IL-6, MCP-1, IL-1b, and IL-10, by macrophages in mice [23]. Decreased SIRT1 expression was found in an HO mouse model one week after burn/tenotomy. Subsequently, the activation of SIRT1 by an agonist inhibited HO progression during the inflammatory phase [23]. The molecular mechanism by which SIRT1 regulates immune responses after injury was associated with downstream NF-κB signalling [9].. These findings suggest the negative role of SIRT1 in regulating NF-κB signalling.

Dysfunctional SIRT1/NF-κB signalling occurs during the transition from monocytes to macrophages and further promotes M1 and M2 polarization and infiltration to injury sites [23]. The depletion of the M1 subtype during the early phase of bone repair can result in the inhibition ossification in soft tissue and the mineralization of ectopic bones, while M2 polarization can commit stem cells to differentiate into the osteoblast lineage [23, 30, 31]. Interestingly, mast cell activation and infiltration are also involved in mediating inflammatory responses involving macrophages. Mast cell activation also occurs through SIRT1/NF-κB signalling. This finding indicates that the role of dysregulated NF-κB-p65 expression in macrophages and mast cells involves the proinflammatory factors that are detrimental to endochondral ossification [16, 23, 32]. Pharmacological repression of canonical NF-κB signalling abrogates HO in rat burn/tenotomy models, and it has been confirmed that overactivation of NF-κB signalling caused by the depletion of SIRT expression is responsible for HO progression [23, 33].

#### AMPK

AMPK is an energy sensor that is intracellularly activated by energy deficiency, such as glucose depletion and hypoxia [34, 35]. AMPK has been shown to play anti-inflammatory roles through the Janus kinase/signal transducer and activator of transcription (JAK/STAT), mitogen-activated protein kinase (MAPK), and NF-κB signalling pathways [36, 37]. A previous study showed that NF-κB signalling/MAPK activation was responsible for ACVR1-mediated inflammation in FOP [16].

Phosphorylated NF-κB p65 and phospho-IKBαSer32/36 were dramatically upregulated in injury sites in HO model mice 3 days and one week after trauma, which indicates dysregulation of the NF-κB signalling pathway in the early stage of HO [8]. Additionally, the decrease of AMPK activity was exacerbated after injury, and there was increased secretion of inflammatory mediators, including TNF-a, IL-6, MCP-1, IL-1β, and IL-10. These findings indicate that a decrease in AMPK activation can coordinate with NF-κB signalling to promote inflammatory macrophage polarization (M1) [8]. Furthermore, the molecular mechanism underlying the inhibitory effect of metformin on HO-related macrophages was examined and showed that metformin could activate AMPK-mediated signalling, which in turn suppressed NF-κB signalling, thereby alleviating HO [8]. Another study on the mechanism of metformin revealed that AMPK activation by metformin could inhibit signal transducer and activator of transcription (STAT3) activity, which promotes monocyte-to-macrophage differentiation [38]. These results further demonstrated that a decrease in AMPK activation could act as a switch to disinhibit the NF-κB pathway, thereby promoting macrophage infiltration and activation. As a result, further secretion of inflammatory mediators, including TNF-α, IL-6 and other chemokines, forms a microenvironment that is conducive to HO. Pharmacological activation of AMPK (e.g., metformin) could inhibit NF-κB signalling in macrophages to further limit their activation, infiltration and inflammatory cytokine secretion, suggesting that therapies targeting AMPK-NF-κB signalling crosstalk are promising for HO patients [8].

#### The NLRP3 inflammasome

Among the various inflammasomes, the NLRP3 inflammasome is the most widely studied and is composed of ASC, NLRP3, NIMA-related kinase 7 (NEK7) and pro-caspase-1 [9]. Recently, a study examined the effect of the NLRP3 inflammasome on macrophage-mediated inflammation in the context of HO [25]. In this study, Li et al. reported that macrophage pyroptosis occurred during HO through an inflammation-driven process, as indicated by significantly increased levels of pyroptosis‐associated molecules, including NOD‐like receptor protein 3 (NLRP3), caspase‐1, gasdermin D (GSDMD) and F4/80 (macrophage marker), 3 and 7 days after tenotomy in mice with HO [25]. Since NF-κB is the central regulator of NLRP3 inflammasome activation, this inflammatory mechanism in macrophages might involve activating the transcription of pro-IL-1β and the NLRP3 gene, which contains NF-κB binding sites within their promoter regions [9, 39]. The HO-induced inflammatory environment contains various inflammatory cytokines that activate TAK1. TAK1 is the inducer that phosphorylates IKK, which further promotes IKβ degradation to allow nuclear translocation, after which NF-κB enters the nucleus to mediate the ubiquitylation of NLRP3, which facilitates NLRP3 inflammasome activation and the protein expression of pro-IL-1β. The active NLRP3 inflammasome, which contains ASC, could promote the production of active caspase 1, which was consistent with previous molecular biomarker results [9, 25]. In addition, active caspase-1 can convert pro-IL-1β to mature IL-1β, which is further secreted from macrophages [9]. Other studies have shown increased IL-1β expression in macrophages after traumatic HO [8, 23]. Interestingly, these secreted inflammatory cytokines are further spread to active neighbouring macrophages, which produce more chemokines to amplify inflammatory responses by establishing positive feedback. Furthermore, the accumulation of IL-1β is important for activating further ectopic bone formation by inducing MSC differentiation [9, 40].

### NF-κB signalling-mediated crosstalk/interactions between macrophages and MSCs

Macrophage-mediated inflammation is the first step in this process, and subsequent MSC differentiation is the key initiator of ectopic bone formation, since HO is an abnormal osteochondral repair process in response to injury [1]. Macrophages are the principal mediators that modulate inflammatory responses, and NF-κB signalling plays an important role in the process by which macrophage-related activities can induce MSC differentiation [25]. The expression of pro-osteogenic genes, such as SRY-box 9 (Sox9) and Collagen Type II Alpha 1 Chain (Col2a1), are induced by inflammatory responses, and typical inflammatory cytokines, including IL-12, IL-3, IL-13 and p70, IL-1 and TNF-α, are considered ligands that activate NF-κB signalling-related receptors on MSCs or macrophages [33, 41, 42]. Activated NF-κB signalling promotes MSC differentiation and chondrogenesis and continues to increase proinflammatory cytokine secretion by macrophages, as well as their activation, polarization, infiltration and proliferation [8, 43].

**Fig. 3:**
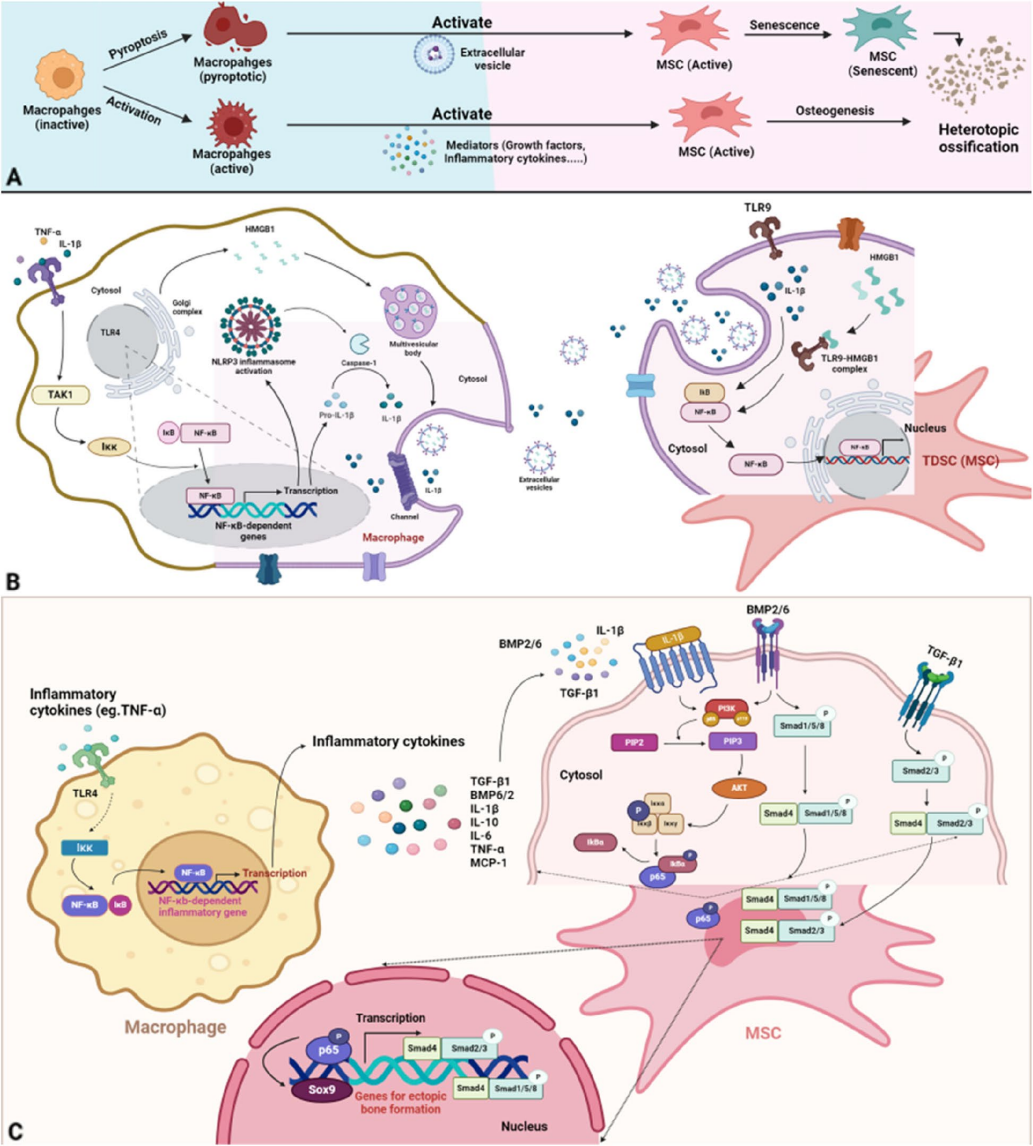
Schematic of NF-κB signalling in the communication between active macrophages and MSCs. **A** General process of HO formation starting from macrophage activation or pyroptosis and MSC activation or senescence leading to ectopic bone formation. **B** Communication between pyroptotic macrophages and TDSCs (MSCs). In macrophages, inflammatory cytokines bind to TLR4, activating IKK and allowing NF-κB to promote NLRP3 and pro-IL-1β gene expression. HMGB1 is packaged in the Golgi and, along with IL-1β, is released in extracellular vesicles. HMGB1-TLR9 complex forms on TDSCs, activating IκB-NF-κB, initiating TDSC osteogenesis. **C** Communication between active macrophages and MSCs. Similar to (**B**), NF-κB-dependent gene expression leads to inflammatory cytokine secretion. IL-1β and BMP2/6 activate PI3K/AKT signalling, promoting P65 translocation into the nucleus for chondrogenic gene expression. BMP and TGF-β induce smad activity for chondrogenesis and osteogenesis-permissive gene expression through smad1/5/8 or smad2/3 and smad4 complex formation (Created with BioRender.com)

The crosstalk between macrophages and osteoprogenitors accounts for aberrant tissue healing through osteogenesis-associated behaviour after traumatic injury [25, 44]. NF-κB signalling has been shown to promote close crosstalk between macrophages and chondrogenic or osteogenic progenitors [25]. In the following section, we will specifically examine how the NLRP3 inflammasome-related inflammatory cytokine IL-1β, high mobility group protein 1 (HMGB1) and BMP signalling mediate the crosstalk between macrophages and osteogenic progenitors (Fig. 3).

#### NF-κB signalling mediates the association via IL-β and HMGB1

In macrophages, the NLRP3 inflammasome was shown to play an inflammatory role, and NF-κB might be involved in inflammatory cytokine secretion. This study also showed a novel crosstalk between pyroptotic macrophages and MSCs, especially tendon-derived stem cells (TDSCs), during HO [25].

MSC senescence after tissue injury significantly impairs the regeneration of injured tissue and results in a false commitment to cell differentiation, causing an osteogenic-lipogenic imbalance [40]. Senescent TDSCs can impair tissue homeostasis by reprogramming tissue healing and releasing factors associated with the senescence-associated secretory phenotype (SASP) [25, 45–47].

The NLRP3 inflammasome facilitates the conversion of pro-IL-1β to mature IL-1β by promoting caspase-1 activity in macrophages [8]. Studies have shown that p16 + TDSCs prominently accumulate in injured tissue, especially near macrophages, and IL-1β, which is released by pyroptotic macrophages is considered to be a key trigger of TDSC senescence and an inducer of TDSC osteogenesis in the inflammatory microenvironment of HO [25]. IL-1β secreted by macrophages can induce the NF-κB signalling pathway in the cytoplasm through the phosphorylation of IKB and the transfer of NF-κB to the nucleus to promote osteogenic and senescent gene expression [8]. HMGB1, which is a damage-associated molecular pattern (DAMP), was shown to be enriched in extracellular vesicles (EVs), and HMGB1 underwent nuclear gene expression and Golgi-mediated modification to form early endosomes, multivesicular bodies and, ultimately, EVs [8]. In addition to IL-1β-mediated NF-κB signalling activation in TDSCs, HMGB1 secreted by pyroptotic macrophages can also induce osteogenesis in an NF-κB-dependent manner [25, 48]. HMGB1 can bind to TLR9 on TDSCs to form a complex that coordinates with pyroM phi-EVs to integrate DNA and is necessary for activating downstream NF-κB signal transduction; IKB is phosphorylated and ubiquitylated for further degradation, allowing NF-κB to be transferred into the nucleus to induce NF-κB-dependent transcription of senescence- and osteogenesis-related genes [8]. Immunohistochemical staining for HMGB1, TLR9 and phosphorylated NF-κb-p65 was increased compared to that in normal tissues, confirming this process. The evidence of TDSC osteogenesis was obtained by coculturing pyroptotic macrophages with TDSCs, followed by alizarin red S (ARS) staining and alkaline phosphatase (ALP) staining [25].

#### NF-κB signalling mediates the association via BMP signalling

BMP is present in the extracellular matrix of bone and is physiologically important in bone morphogenesis [49]. Pathologically, BMP signalling plays an important role in HO [50]. Many BMP ligands, including BMP2/4/7/9, have been shown to induce HO and are highly secreted after damage to soft tissue [51–53]. In FOP, which is the inherited type of HO, the causative gain-of-function of ACVR1 could result in the overactivation of BMP signalling and contribute to HO [54, 55]. BMP expression is enhanced in response to inflammation, and BMP is secreted into injured soft tissues after injury [56], which indicates a link between inflammatory stimuli and osteogenic differentiation.

Overactivation of the NF-κB pathway in M1 and M2 macrophages can induce the expression of BMP6 and strengthen the BMP signalling pathway to increase osteogenic differentiation [8]. NF-κB-induced BMP signalling further promotes the transcription of BMP-related genes, including the downstream molecules smad1/5, and the phosphorylation of smad1/5 plays a key role in bone formation and repair, especially during HO pathogenesis. Research has shown that coculturing macrophages and osteoblast progenitor cells leads to additional NF-κB signalling activation in macrophages, which can promote the secretion of BMP6, BMP2 and TGF-β1, which in turn activates osteogenesis and promotes osteoblast differentiation [8, 57]. This evidence confirms that NF-κB signalling in macrophages can further stimulate BMP signalling in MSCs or osteoblasts to promote their differentiation and bone maturation through cytokine secretion. 

### NF-κB signalling in chondrogenesis

Interestingly, it seems that the chondrogenic development during HO strongly simulates endochondral ossification, which suggests that the molecular machinery in MSCs and chondrocytes under normal conditions can provide many clues about pathological bone formation [58, 59]. Therefore, we can theorize that the chondrogenesis stage of HO is based on a situation in which partial natural bone formation is achieved by chaotic molecular signalling to some extent but occurs at an abnormal anatomic site, such as soft tissue. These cellular activities are, however, temporally and spatially disorganized in HO. As a result, the tissues are chaotic and uneven during HO. Additionally, mature heterotopic bone can create bone marrow cavities and generate bone marrow that appears to be normal [58, 59].

In pathological bone, the process of endochondral ossification involves a number of steps, including the condensation of mesenchymal cells, the differentiation of MSCs into chondrocytes, chondrocyte proliferation, hypertrophy, apoptosis, vascular invasion, and calcification [60, 61]. These complex programs are constantly and expertly controlled during development. 

**Fig. 4:**
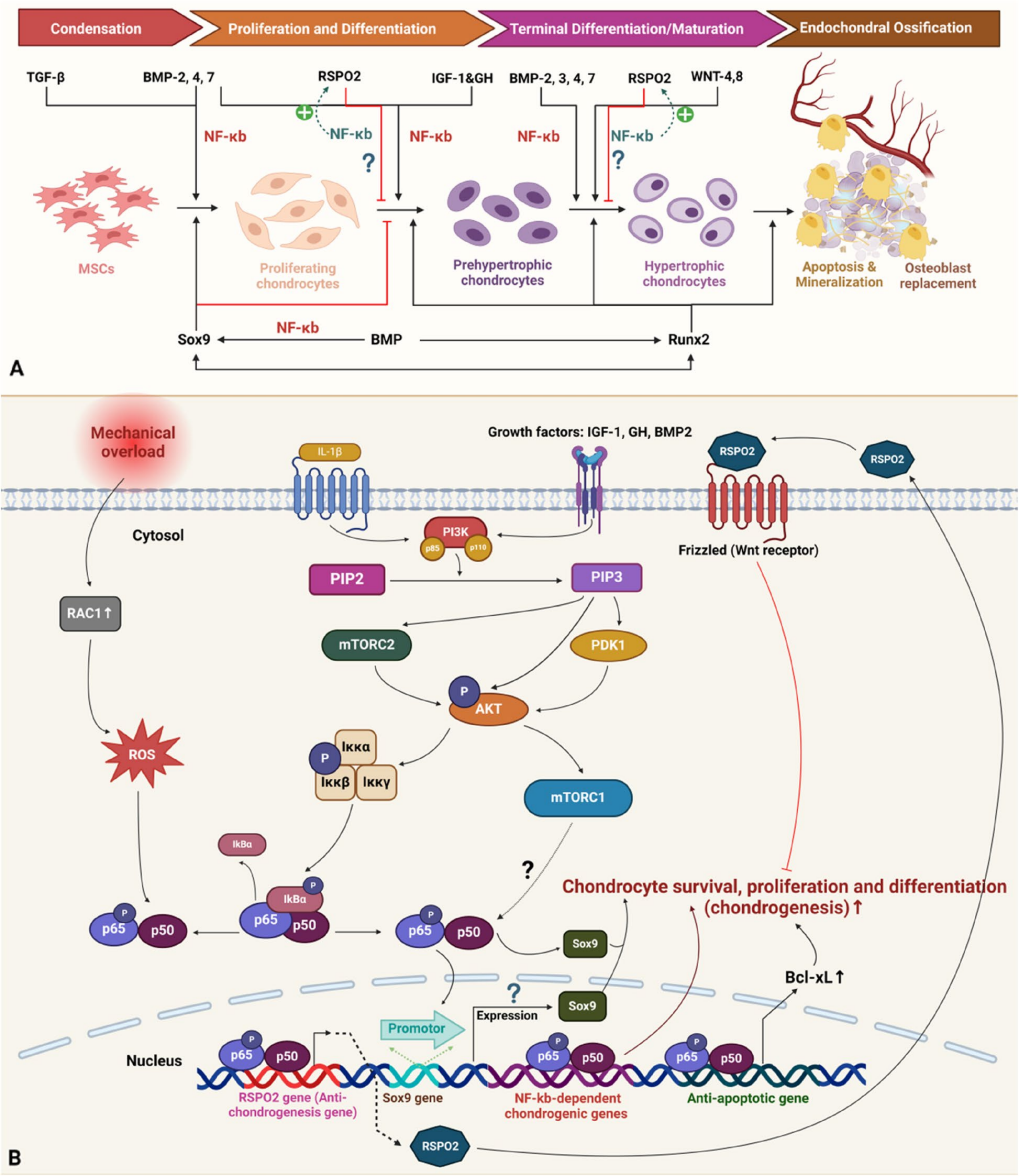
Schematic of NF-κB signalling during chondrogenesis in endochondral ossification. **A** General scheme of chondrogenesis and related molecules and signalling pathways. I. Condensation: MSC condenses into proliferating chondrocytes. TGF-β and BMP signalling regulate this stage, with BMP positively modulating NF-κB signalling. II. Proliferation and Differentiation: Proliferating chondrocytes transition to prehypertrophic chondrocytes. BMP and IGF-1/GH positively regulate NF-κB signalling, while RSPO2 counteracts it, with reduced expression during this stage. III. Terminal Differentiation: Prehypertrophic chondrocytes mature into hypertrophic chondrocytes. BMP and Wnt signalling positively regulate NF-κB signalling, while RSPO2 antagonizes it. Sox9 expression is regulated by NF-κB and is present in proliferating chondrocytes but suppresses their differentiation into prehypertrophic chondrocytes and is absent in the final maturation stage. **B** Detailed molecular mechanism of NF-κB regulation during chondrogenesis. Inflammatory macrophage-derived IL-1β and growth factors (IGF-1, GH, BMP2) activate PI3K/AKT signalling. PI3K converts phosphatidylinositol - 4,5 - bisphosphate (PIP2) into phosphatidylinositol [3–5]-trisphosphate (PIP3), activating AKT via mTORC2 and pyruvate dehydrogenase kinase 1 (PDK1). mTORC1 signalling may also stimulate NF-κB-65. Active P50/P65 translocates to the nucleus, inducing Sox9 promoter activity, chondrogenic gene expression, and B-cell lymphoma-extra large (Bcl-XL) expression for chondrocyte survival, proliferation, and differentiation. Mechanical overload stimulates Rac1, activating NF-κB signalling to promote RSPO2 expression, which inhibits chondrogenesis (Created with BioRender.com)

NF-κB signalling could promote MSC differentiation into chondrocytes and subsequent chondrocyte survival, proliferation and differentiation [62–65]. These events were shown to be partly mediated by interactions with other signalling pathways, including PI3K/AKT signalling, which is induced by growth factors, including IGF-1 and BMP2, as well as mechanistic target of rapamycin complex 1 (mTORC1) signalling [62, 66–70]. In addition, active NF-κB signalling can regulate other molecules, including Sox9 and Rspo2. Sox9 expression is activated by NF-κB induction, and NF-κB-p65 can induce the expression of Rspo2, which inhibits chondrocyte development [64, 65] (Fig. 4).

#### Growth factors: IGF-1 and BMP2

IGF-1 has been proven to induce NF activation, which further regulates chondrogenesis in growth plates by promoting the proliferation and maturation of chondrocytes [71, 72]. Similarly, growth hormone (GH) can stimulate chondrogenesis in the growth plate and bone growth [73]. The underlying mechanism involves increasing NF-κB, BMP2 and p65 mRNA expression and the phosphorylation of proteins in the growth plate [73]. IGF-1 was proven to activate NF-κB signalling specifically in chondrocytes in the growth plate, and PDTC (an NF-κB inhibitor) could reverse or abrogate the positive effects of IGF-1 on cell division in the proliferative and epiphyseal zones of the plantar growth plate and IGF-1-mediated stimulation of longitudinal expansion of the metatarsal. Additionally, a study showed that suppressing Akt and PI3K could prevent IGF-1-induced NF-κB-p65 activity in chondrocytes [73]. Further investigation showed that IGF-1 could activate PI3K to enhance Col2a1 expression, and wortmannin, which is a PI3K inhibitor, significantly reduced the effect of IGF-1 on Col2a1 expression [74]. Interestingly, a study used exosomal miR-140-5p to target IGF1R and inhibit the differentiation of human MSCs by decreasing the phosphorylation of the insulin receptor substrate (IRS1)/PI3K/AKT/mTOR pathway [63]. This evidence demonstrated that IGF-1 could stimulate longitudinal bone development and all of the key cellular processes involved in chondrogenesis in the growth plate by at least partially activating NF-κb-p65 through the PI3K/Akt pathway [71]. Additionally, the developmental effects of GH are mediated by p65, according to a report in which 2 different mutations impairing NF-κB induction in 2 children resulted in growth deficiency and GH tolerance [71]. In contrast, patients with acromegaly suffer from ossification of the posterior longitudinal ligament as a result of excessive secretion of GH and IGF-1 [75]. These findings confirmed that GH and IGF-1 could promote additional chondrogenesis for final ectopic bone ossification in soft tissue, and NF-κB signalling might be involved in this process.

Furthermore, p65 overexpression can induce overactive chondrocyte differentiation and proliferation, which is accompanied by antiapoptotic effects such as an increase in BMP2 expression [19]. Conversely, suppressing NF-κB signalling with BAY11-7082, PDTC or a short interfering RNA (siRNA) targeting p65 could reduce the differentiation and proliferation of chondrocytes [71, 72]. A study in chicken embryos revealed that overexpression of IκBα, which is a dominant negative form, could prevent NF-κB activation, ultimately downregulating BMP signalling and abnormal limb bud development [19]. Furthermore, the molecular mechanism underlying the activation of NF-κB signalling by BMP was examined, and BMP2 was shown to trigger NF-κB via AKT/PI3K, which was also reported to promote chondrogenesis in the growth plate; moreover, evidence has shown that the association between NF-κB and PI3K/AKT involves the phosphorylation of p65 at Ser-536 by IKK [66–70]. Additionally, there are 2 NF-κB responsive elements in the promoter region of the BMP2 gene [76]. Experiments based on NF-κB2 and NF-κB1 double-knockout mice showed a profound reduction in BMP2 expression in chondrocytes in the growth plate, which confirmed that NF-κB could also activate BMP signalling [76]. These findings indicate a synergistic relationship between BMP and NF-κB signalling that cooperate with each other during chondrogenesis, which might be essential for HO during aberrant chondrocyte proliferation and further differentiation in ectopic soft tissues. Moreover, a study on the pathogenesis of osteoarthritis suggested that PI3K/AKT could alleviate the degeneration of cartilage by inhibiting chondrocyte apoptosis partially via NF-κB signalling [77]. This evidence provides insight into the pathogenesis of HO, and overactive NF-κB signalling might contribute to abnormal chondrocyte accumulation [19, 76, 77].

#### mTORC1

In the context of endochondral ossification, mTORC1 expression and effects are dynamically altered during osteogenesis, particularly during the chondrogenesis stages, and a study showed that mTORC1 plays different roles in different stages of HO [78]. During ectopic cartilage formation, mTORC1 signalling was activated during the first 2 weeks after burn/tenotomy and was highly expressed 4 weeks after traumatic stimuli [62]. However, these levels were low in normal tendon tissue where ectopic cartilage maturation occurred. The use of rapamycin, which is as an mTORC1 inhibitor, could inhibit chondrogenic differentiation in tendon cells but was later shown to activate chondrocyte maturation and hypertrophy. Interestingly, mTORC1 activation has been shown to exert opposite effects or phage-specific effect during the progression of chondrogenesis [62]. This phenomenon was similar to physiological cartilage development, and tendon stem cells changed properties by undergoing cross-differentiation into chondrocytes, which was driven by high levels of mTORC1 signalling, and low expression of mTORC1 was found during the final maturation stage of chondrocytes, including cellular hypertrophy, further degradation and the gradual mineralization of the cartilage matrix, preparing for replacement by osteoblasts to mediate bone formation [78].

In the chondrogenic differentiation stage, mTORC1 signalling was reported to activate cartilage gene transcription via the NF-κB-p65 pathway, and p65 activity is crucial for chondrogenesis [19, 62]. Evidence suggests that in vitro activation of mTORC1 signalling could increase the nuclear translocation of p65 in the absence of an NF-κB activator [62, 78]. This finding indicates that the positive interaction between mTORC1 and NF-κB has an upstream–downstream molecular relationship. However, because rapamycin can block chondrogenic differentiation, BAY-11-7082 (an Iκκ inhibitor) was also shown to rescue tendon stem cell differentiation, and mTORC1 signalling and NF-κB signalling play similar roles in driving chondrogenesis. The effects of NF-κB and mTORC1 may be associated with Sox9-mediated promotion of chondrogenic gene expression [78]. Sox9 expression is increased at the mRNA and protein levels by excessive mTORC1 activation, which supports chondrogenesis in tendon stem cells [62]. Sox9 is a downstream signal of NF-κb-p65 [79]. In proliferating chondrocytes, Sox9 expression is high, and its transactivation target, Col2a1, is actively transcribed to form fundamental collagen in the extracellular matrix, while Runx2 expression is inhibited [65]. This effect can be induced by high levels of mTORC1 signalling and NF-κB signalling. When the prehypertrophic and hypertrophic chondrocyte stages are reached, mTORC1 signalling decreases, which might also result in decreased NF-κB expression. A study showed reduced Sox9 activity during these 2 stages, which might indicate reduced NF-κB activity [80]. In summary, during the chondrogenesis stage of HO, mTORC1 signalling was increased in tendon cells in which excessive NF-κB activity was triggered to mediate chondrogenesis, and a selective inhibitor of NF-κB, such as BAY11-7082, could inhibit TD chondrogenesis during mTORC1-induced HO progression [62].

#### SOX9

SOX9, which is a key transcription factor, controls a variety of chondrogenesis-related processes [81, 82]. It is a member of the SRY-related high-mobility group (HMG) box (SOX) family of transcription factors, which are crucial for selecting cell fate, including dedication to chondrogenesis [82, 83]. Sox9 is a downstream signal of NF-κb-p65 [33, 79, 84]..Interestingly, NF-κB seems to have regulatory effects on Sox9 and indirectly regulates chondrogenesis during HO.

A study revealed that RelA (NF-κB-p65) could significantly activate the human Sox9 promoter [85], and Sox9 expression was thought to promote chondrogenesis of tendon cells. Similarly, RelA mediates the complex molecular network involved in transactivation rather than acting as a main transactivator of Sox9, which indicates that the activation of RelA signalling is not sufficient to fully induce Sox9 activation or adequate chondrogenic differentiation [85]. Additionally, a previous study revealed that TNF-α and IL-1, which have deleterious effects, could suppress Sox9 expression in chondrogenic cells, which might be associated with pathological degenerative cartilage diseases, including osteoarthritis [86]. These findings indicate that inflammatory cytokines can induce Sox9 inhibition partially via NF-κB signalling. Curcumin has been proven to inhibit osteoarthritis, and the underlying mechanism involves the inhibition of p65-NF-κB activity in the nucleus, which results in the disinhibition of Sox9 expression to promote chondrocyte differentiation [87]. Interestingly, the suppressive effect of NF-κB on Sox9 regulation seems to contradict the positive relationship between these two proteins, especially in the context of HO, and macrophage-secreted inflammatory cytokines tend to induce Sox9 expression to promote chondrogenesis [33]. This finding might also be consistent with the opposite outcomes observed in HO and osteoarthritis, such as ectopic bone formation and bone degeneration. Normally, during the first few hours of chondrogenic differentiation, BMP2 induces brief activation of NF-κB, and p65 siRNA inhibits BMP2-induced Sox9 expression in ATDC5 cells, showing that BMP2 induces Sox9 expression via NF-κB activation during the early chondrogenic phase of endochondral ossification [84]. However, additional research might be needed to determine additional associations between Sox9 and NF-κB in HO to determine additional clues about the difference from osteoarthritis, since these conditions have similar pathophysiological backgrounds in terms of the molecular signalling pathways (AKT/PI3K) involved in chondrocyte differentiation but different pathologies. However, overactive NF-κB signalling in chondrocytes can disturb physiological bone development and form ectopic bones. [79, 84].

#### RSPO2

Proteoglycan 4 (PRG4), which is commonly known as lubricin, is typically expressed as an extracellular matrix component in the synovium, tendons, ligaments and articular cartilage [88, 89] and lubricates the boundaries of joints and tendons [88]. Human PRG4 loss-of-function mutations cause camptodactyly arthropathy-coxa vara-pericarditis syndrome [90]. Examination of the characteristics of Prg4-positive ( +) cells revealed that RSPO2, which is a WNT activator, is specifically expressed in the Prg4 + TSPC cluster. In recent years, lubricin has been shown to be expressed in combination with RSPO2, which is a feature of undifferentiated tendon stem/progenitor cells (TSPCs) [64]. A study established a mouse HO model and examine the role of RSPO2 in OPLL and the underlying mechanism. Endochondral ossification occurred in mice after Achilles tendon puncture, and this process was inhibited by RSPO2 [64]. Ossification of the posterior longitudinal ligament (OPLL) is an atypical type of traumatic HO that mainly occurs at the spine and posterior longitudinal ligament [91, 92].

Since RSPO2 is known as the susceptibility gene for OPLL and is downregulated in OPLL patients, further studies have shown that high expression of NF-κB is induced to precipitate RSPO2, which can be triggered by inflammation or mechanical overload of ligaments or tendons [64]. In the context of inflammation, human ligament cells cultured with IL-1β exhibited marked RSPO2 induction, the IKK inhibitor Bay11-7085 suppressed this induction, and the RSPO2 + cluster showed high RelA mRNA expression [64]. Mechanical stimuli can induce marked RSPO2 expression, and RSPO2 protein levels are proportional to mechanical overloading, which is accompanied by increased inhibitor of IκBα phosphorylation. The Rac1 inhibitor EHT1864 could reduce RSPO2 induction by mechanical overload. In brief, mechanical loading and inflammation-induced RSPO2 expression occur through the Ras-related C3 botulinum toxin substrate 1 (Rac1)-NF-κB axis; NF-κB acts as an upstream regulator, while RSPO2 is located downstream [64, 93]. Repeated mechanical overloading or inflammatory conditions can lead to degeneration and improper ossification of the ligament or tendon [64]. By inhibiting chondrogenic development in progenitor cells, the Rspo2 + cluster can aid in proper maintenance or healing of the tendon/ligament under these pathological conditions [64, 94]. In other words, an excessive mechanical load can trigger HO in tendon cells, and a negative regulator antagonizes this driving force by upregulating RSPO2 expression. Furthermore, RSPO2 can be transported extracellularly and bind to the WNT receptor to inhibit chondrogenesis, restoring the balance of bone formation [64, 95]. Therefore, therapeutics targeting RSPO2 expression might reverse ectopic ossification in tendons.

### NF-κB signalling in osteogenesis

**Fig. 5:**
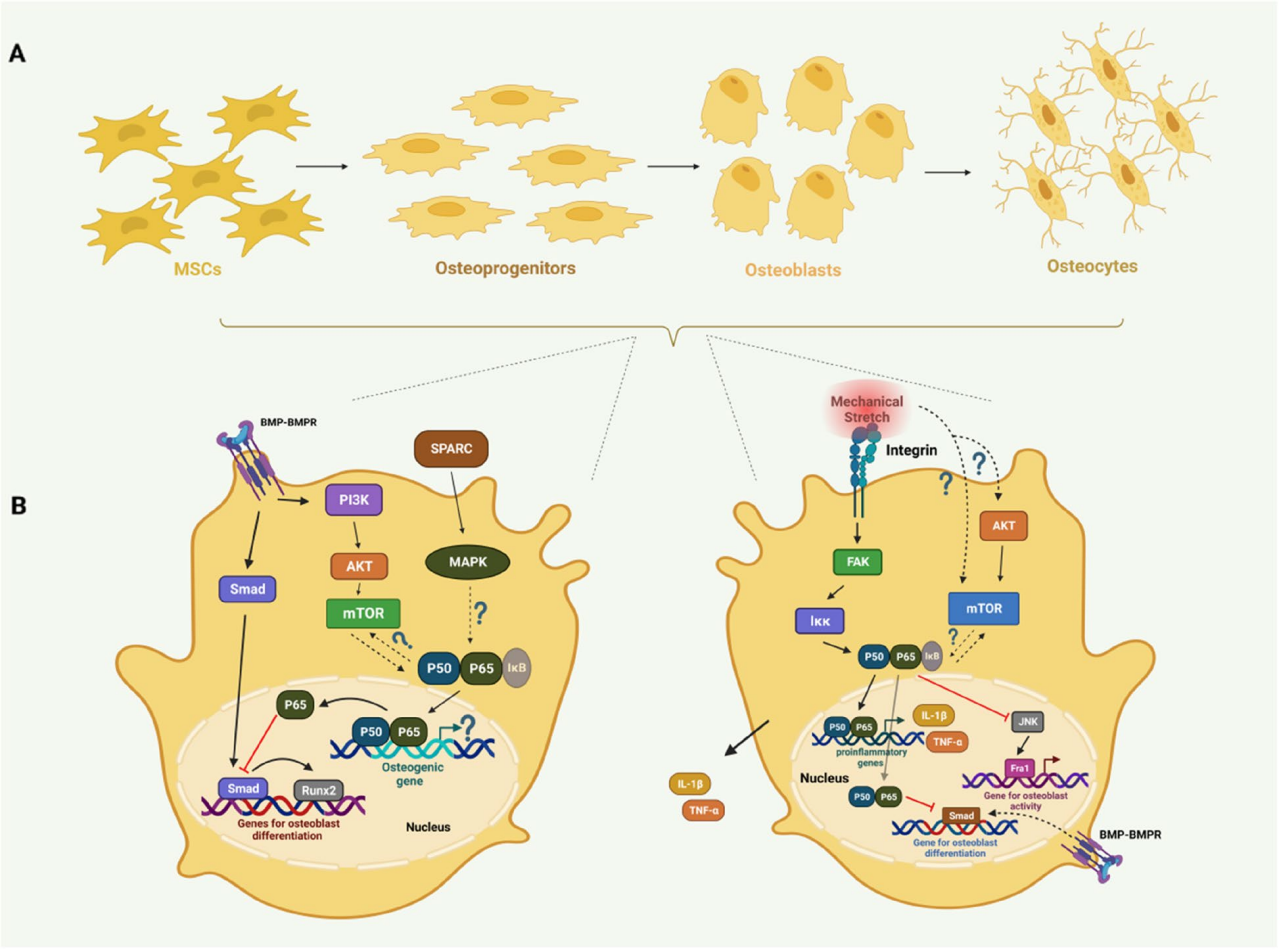
Possible schematic diagram of NF-κB signalling during osteogenesis of endochondral ossification. **A** Macroscopic scheme of the general process of osteogenesis. MSCs condense into osteoprogenitors. Then, osteoprogenitors differentiate into osteoblasts. Finally, osteoblasts mature into osteocytes. **B** The left osteoblast: During osteogenesis, SPARC expression is decreased and can activate the phosphorylation of MAPK, which might induce NF-κB signalling to initiate osteogenic expression. However, on the one hand, BMP could promote osteoblast differentiation via smad signalling. On the other hand, BMP-mediated signalling may promote NF-κB activity via PI3K/AKT/mTOR axis where P65 may inhibit smad signalling to induce Runx2 expression and activity to block osteoblast differentiation. The right osteoblast: mechanical stress may activate mTOR signalling via AKT activation or integrin-mediated FAK signalling to further induce P50/P65 which could promote proinflammatory gene expression including IL-1β and TNF-α. Additionally, P50/P65 could also inhibit smad activity induced by BMP signalling to decrease osteoblast differentiation. Moreover, active P65 may inhibit JNK which could further repress Fra1 to dampen osteoblast activity (Created with BioRender.com)

The osteogenesis stage is the latter stage of HO, during which hypertrophic chondrocytes undergo apoptosis, allowing osteoblasts to invade the empty spaces between extracellular spaces; these cells further differentiate into osteocytes, which leads to further ossification in non-osseous tissue [59, 96]. NF-κB signalling is vital for regulating osteoclast differentiation and activation [3]. Recently, NF-κB signalling was proven to inhibit osteoblast differentiation and osteogenesis, which indicates that its inhibition permits osteocyte formation [23, 32, 97]. As a result, NF-κB was thought to be important for connecting bone production and bone resorption and ultimately for maintaining the proper equilibrium of bone remodelling [98]. Overall, the involvement of NF-κB signalling during osteogenesis was shown to predominantly inhibit osteoblast differentiation [97, 99]. In terms of the regulation of NF-κB signalling, mTOR signalling can positively induce NF-κB signalling to modulate osteogenesis in response to mechanical stimuli [97]. Moreover, apart from the mTOR-NF-κB axis, integrin-mediated NF-κB signalling activation could inhibit osteoblast differentiation [99]. Additionally, BMP signalling was also shown to be involved in osteogenic regulation via its inhibitory interaction with NF-κB signalling and synergistic interaction with mTOR signalling [100–102]. However, another study revealed that the protein expression of secreted protein acidic and rich in cysteine (SPARC) was upregulated during HO, which could promote NF-κB phosphorylation to activate osteoblast differentiation [103] (Fig. 5).

#### SPARC

SPARC, which is also known as Osteonectin, is thought to play a role in the survival, formation and maturation of osteoblasts [103]. Similarly, SPARC can also act as an indicator of bone formation and has the ability to promote osteoblast differentiation and calcium deposition [99, 104]. A previous study demonstrated that receptor activator for nuclear factor-κB ligand (RANKL) could activate Pi-induced calcification of cardiac fibroblasts by activating SPARC in vivo [105]. RANKL-RANK is the classical pathway mediated by NF-κB that indicates a relationship between NF-κB and SPARC to promote chondrogenic ossification [99, 103, 106]. 

Physiologically, ligament fibres and the periosteal epithelium at the tip of the styloid process exhibit strong SPARC positivity [107]. Pathologically, research has shown that SPARC expression is strongly increased in HO tissues [103, 106]. SPARC is capable of increasing the phosphorylation of mitogen-activated protein kinase 2 (MAPK-APPK2) and p38 MAPK and can activate p38 MAPK-heat shock protein 27 (HSP27) signalling [108]. In traumatic HO patient serum, the MAPK signalling pathway was activated to promote osteogenic differentiation in adipose tissue-derived stem cells [109]. Interestingly, ACVR1 was shown to positively potentiate NF-κB and p38/MAPK activity, leading to an inflammatory environment. This is the mechanism of ACVR1-dependent inflammation in inherited HO (FOP) [16]. Therefore, high expression of SPARC in the rat tenotomy/burn model could activate NF-κB signalling and the MAPK pathway to promote HO.

The MAPK signalling pathway was strongly increased by SPARC activation in an HO rat model. Following MAPK activation, NF-κB and Iκκβ phosphorylation are markedly enhanced after SPARC activation [103]. NF-κB can stimulate activator protein 1 to activate MAPK and further promote pathological bone formation [106, 110]. Additionally, similar to mTORC1, Akt is important for mediating NF-κB signalling and is controlled by mTOR and Raptor, which are related to Iκκ [111]. Runx1 interacts with Iκκ in the cytoplasm to mediate the NF-κB signalling pathway [112]. However, suppressing SPARC could inhibit MAPK signalling, further inhibiting NF-κB signalling to alleviate HO progression [106].

#### Mechanical stress involving mTOR and integrin

During chondrogenesis, mTORC1 signalling has been proven to promote chondrogenic differentiation in tendon-derived stem cells, and this effect is decreased when chondrocytes become mature and hypertrophic [62]. Furthermore, mTOR signalling was shown to play a role in osteoblast differentiation [97]. In vitro and in vivo, mechanical stretching was shown to enhance the osteoblast phenotype and promote osteogenesis [97]. The mTOR pathway was shown to be stimulated by cyclic mechanical strain, and NF-κB p65 phosphorylation and nuclear translocation were also promoted. Inhibition of NF-κB with PDTC or siRNA enhanced the expression of genes linked to osteogenesis, and inhibition of mTOR with rapamycin decreased the expression of genes related to osteogenesis in response to mechanical stretching. Additionally, blocking mTOR signalling reduced the phosphorylation of NF-κB, and inhibiting NF-κB signalling also decreased the activation of mTOR in response to mechanical stretching. Overall, this study showed that mechanical overload could dramatically promote osteogenic differentiation by altering or reshaping the osteoblast phenotype by stimulating osteogenic markers, including ALP, BMP and Runx2, at the early stage as well as Col1a1 at the late stage [97]. This molecular mechanism is different from mTORC1-NF-κB signalling in the chondrogenesis stage, and mTORC1 signalling is first upregulated to promote chondrocyte differentiation but later downregulated to initiate chondrocyte hypertrophy [62]. A study demonstrated that the reciprocal interaction between NF-κB and mTOR signalling in osteoblasts is a crucial response to mechanical stretching [97]. This finding reveals the potential link between the coregulation of mTOR-NF-κB and HO.

Surprisingly, co-inhibition of NF-κB and mTOR led to higher osteogenic gene expression than inhibition of only mTOR but lower osteogenic gene expression than blockade of NF-κB alone. This suggested that there was a reciprocal interaction between mTOR and NF-κB, which indicated that osteoblast differentiation was mediated by the positive regulation of mTOR and negative regulation of NF-κB signalling [97]. In terms of the regulatory relationship between mTOR and NF-κB, mTOR suppression could decrease NF-κB signalling, and NF-κB blockade could reduce mTOR expression in osteoblasts in response to mechanical stretching [97]. In summary, NF-κB functions as a negative regulator of osteoblast development, and mTOR positively regulates stretch-induced osteoblast differentiation. Additionally, when mTOR was inhibited, NF-κB expression was decreased, and when NF-κB was blocked, mTOR expression was reduced in osteoblasts subjected to mechanical strain [97]. Mechanical stretch has been identified as an HO-permissive factor, and calcific tendinopathy overload can induce HO via strong activation of the mTORC1 signalling pathway in tendon cells [95]. Another study revealed that mechanical stretching could stimulate osteogenic differentiation in human bone MSCs by suppressing NF-κB signalling [113]. Similarly to osteoblast differentiation, NF-κb signalling is considered a negative modulator that inhibits differentiation in osteoblasts [114].

Moreover, a study revealed that the inhibition of upstream molecules, such as Akt, could have a similar effect as the co-inhibition of NF-κB and mTOR, which indicates that ectopic bone formation may also be associated with Akt, which is a common upstream regulator [97]. These findings indicate the potential relationship between mTOR and AKT, in which NF-κB might act as a regulator.

Apart from mTOR-induced osteoblast differentiation in response to mechanical stress, integrin-mediated NF-κB signalling in response to mechanical stimuli can partially rescue osteogenesis. Runx2 and its downstream effector osterix (Osx) are the key transcription factors necessary for determination of the osteoblast lineage. When osteoblasts mature, they need to bind tightly to the bone surface using β1 integrins and start secreting matrix proteins in a direct manner. Integrin-mediated signals can facilitate Runx2 transcription and induce the proliferation and biosynthetic response of osteoblasts in response to mechanical pressure. Moreover, TNF-α and IL-1β are downstream NF-κB targets. This suggests that the activation of NF-κB signalling in response to mechanistic induction can promote inflammatory cytokine secretion, and focal adhesion kinase (FAK), which is an essential component of the adhesion complex, can mediate signal transmission downstream of integrin-induced interactions with the extracellular matrix, which is necessary for activating the stress-associated NF-κB pathway in osteoblasts to inhibit their differentiation [99]. Therefore, dysregulated signalling, including that of mTOR and NF-κB, in response to mechanical stimuli can trigger HO. However, to some extent, the inhibitory responses mediated by NF-κB signalling in osteoblasts in response to mechanical stimuli might constitute a native negative feedback mechanism to block HO progression.

#### BMP

BMP signalling is important for activating chondrogenic and osteogenic development [8, 76, 115]. During osteogenesis, BMP4/6/7/8/9 can increase osteogenic activities, thereby contributing to HO progression, and antagonizing BMP signalling can attenuate HO severity [115]. Research has revealed the relationship between BMP signalling and NF-κB signalling in osteoblasts during osteogenesis [99]. Furthermore, SMAD activity is disrupted by NF-κB downstream of BMP2 or TGFβ [99]. Further evidence that p65 can coordinate with the Smad1-Smad5 complex in the nucleus and interfere with its ability to bind to target promoters was provided by Yamazaki et al. [116]. NF-κB signalling can block BMP signalling to downregulate osteoblast differentiation. A study revealed that a decrease in NF-κB activity could disinhibit c-Jun N-terminal kinase (JNK) activity and enhance fos-related antigen (Fra1), which is required for stimulating osteoblast differentiation through NF-κB inhibition [117]. In summary, activation of NF-κB signalling can block osteoblast differentiation during the osteogenesis stage of HO.

Moreover, BMP2 could promote protein synthesis via PI3K/mTORC1 activation in osteoblasts, and an experiment showed that BMP2 could activate the PI3K/AKT axis in 2T3 cells [101]. mTOR activation can promote osteoblast differentiation [97]. These findings indicate the synergistic relationship between mTOR signalling and BMP signalling in osteoblasts. Interestingly, NF-κB is believed to be the downstream effector of mTOR, which indicates that the favourable osteogenic effects of BMP signalling might also be partly mediated by the inhibition of osteogenic gene expression by mTOR-NF-κB signalling [62].

## NF-κB signalling in FOP

### NF-κB signalling in the inflammation stage of FOP

**Fig. 6:**
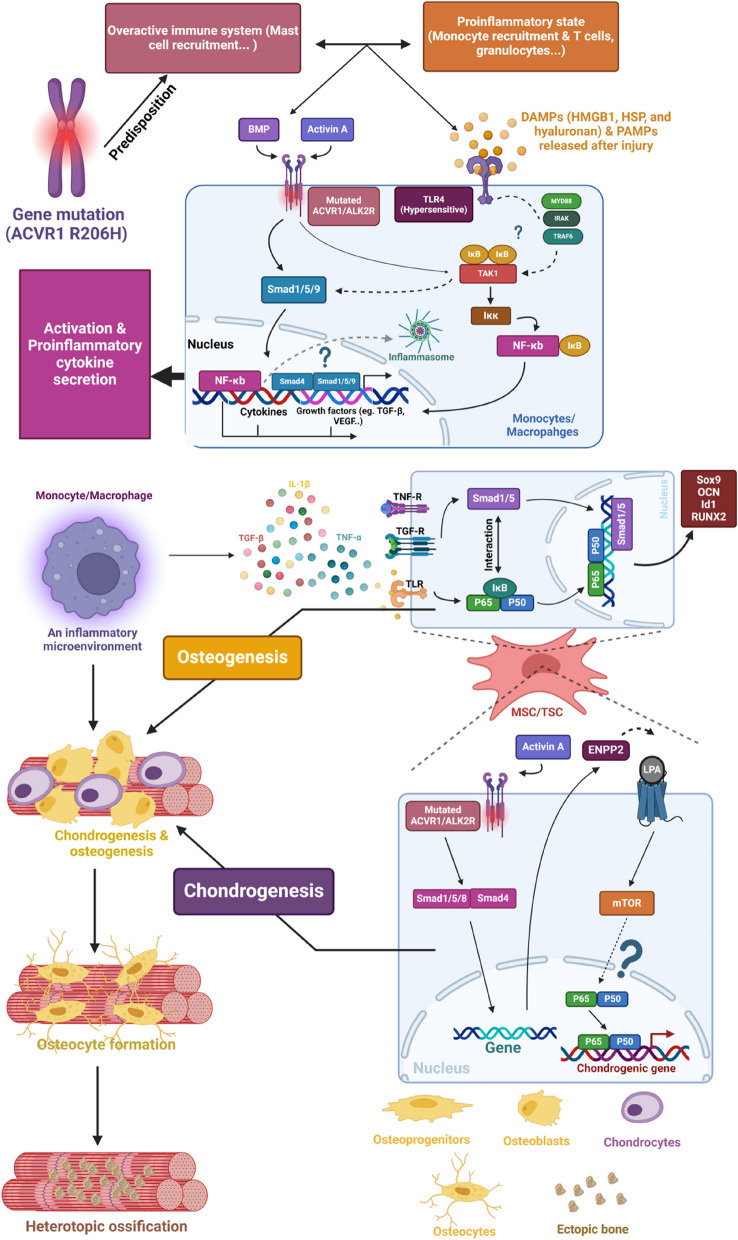
Scheme of FOP pathogenesis and the involvement of NF-κB signalling. FOP is characterized by an autoinflammatory state and immune overactivation in soft tissues, involving immune cells like mast cells, T cells, and monocytes/macrophages. These immune cells create an inflammatory environment, which further activates monocytes/macrophages through the TLR4-related proinflammatory signalling pathway. The ACVR1 R206H mutation enhances the responsiveness of the ALK2 receptor to BMP and activin A, activating the smad signalling pathway and promoting the expression of growth factors, including TGF-β. ACVR1 and TLR4 can also activate TAK1 to promote NF-κB signalling activation, leading to the expression of inflammatory cytokines (TNF-α, IL-1β) and growth factors (VEGF). Inflammasome activation may also contribute to cytokine secretion. Monocyte-secreted molecules like TGF-β and IL-1β recruit MSCs/TSC by activating NF-κB and smad signalling. In MSCs committed to an osteogenic fate, NF-κB and smad synergistically promote osteogenic gene expression (OCN, Sox9, Runx2, Inhibitor of DNA Binding 1 (Id1)). In MSCs committed to a chondrogenic fate, ACVR1 mutation-induced ENPP2 activates mTOR signalling via lysophosphatidic acid (LPA) receptor binding, leading to NF-κB activation and chondrogenesis. Chondrocytes undergo maturation, degradation, and replacement, while osteoblasts differentiate into osteocytes, ultimately resulting in ectopic bone formation (Created with BioRender.com)

NF-κB signalling plays an important role in FOP [16, 97]. As previously stated, ACVR1 R206H causes a genetic predisposition for macrophage hypersensitivity to certain stimuli. After injury, a variety of internal TLR4 DAMP proteins, such as HMGB1, hyaluronan, and HSP, which are vital for regeneration and repair of tissue, are activated [118]. FOP patients can experience a hypersensitive inflammatory response caused by pathogen-associated molecular patterns (PAMPs) or DAMPs generated following injury, which results in the swelling of inflammatory soft tissue during flare-ups of bone production. The effect of ACVR1 R206H on macrophages might be specific to the TLR4 pathway, as evidenced by the lack of enhanced responses observed when TLR2/6 ligands were tested. TLR4 is an important inflammatory receptor for the activation of the NF-κB signalling pathway [9]. Therefore, mutations in TLR4-related machinery can increase inflammatory responses by activating NF-κB signalling [16] (Fig. 6).

Excessive activation of NF-κB has been found in FOP monocytes, and abnormal regulation of TLR-mediated signalling has also been found in progenitor cells in FOP connective tissue [42]. This indicates a potential relationship between hyperresponsive TLR4 activity and an increase in NF-κB signalling in FOP. In addition, the causative factor of the hyperactivity of macrophages, in addition to NF-κB signalling, may be involved in p38-mediated effects on anti-inflammatory macrophages [16]. Virtually all ACVR1 and TLR4 can modulate TAK1 expression, which is favourable for the NF-κB and Smad1/5 pathways [119–121]. Studies have also revealed an aberrant increase in Activin A, which stimulates BMP-like associateresponses [122, 123]. Interestingly, Smad1/5/9 signalling was not significantly altered in FOP macrophages/monocytes [16]. This indicates that other gene-specific mutation-induced pathways are present in macrophages. In addition, TAK1 phosphorylation was increased in FOP and control monocytes, which indicated that the stimulation of TAK1 during the pathogenesis of chronic inflammation may also involve inflammasome activation [16, 124]. Specifically, inflammatory cytokines, including IL-1β and TNF-α, have high binding affinities with TLR4, which further activates downstream myeloid differentiation factor 88 (MyD88), IRAK and TNF receptor–associated factor 6 (TRAF6). TAK1 is activated to phosphorylate IKK, which eventually leads to the nuclear translocation of NF-κB [9, 125]. ACVR1 can alter the expression of TAK1 and further IKK activation [119, 120], allowing nuclear transcription of NF-κB in monocytes [121] and differentiated anti-inflammatory macrophages [119, 120]. ACVR1 phosphorylates Smad1/5/9, which further binds with Smad4 to form a complex in the nucleus to promote gene expression [126]. A study in which tendon stem cells were used as a cellular model of HO revealed that the expression and phosphorylation of p65 increased with Smad overexpression but decreased with the depletion of Smad [127]. These findings indicate that Smad and NF-κB have synergistic effects on coactivation and that in an inflammatory environment, they contribute to the differentiation of osteoblasts to induce HO.

### NF-κB signalling in FOP inflammation and ectopic bone formation

The inflammatory microenvironment is important for driving ectopic bone formation during FOP [16]. Inflammation can induce NF-κB signalling, which can further activate downstream Smad activity. Smad signalling is important for the expression of osteogenic genes, including OCN, Sox9 and Runx2. Osteogenic gene expression can alter MSC fate by accelerating MSC differentiation into preosteoblasts for final ectopic bone formation [128]. Moreover, it has recently been demonstrated that during chondrogenesis triggered by activin A, mesenchymal stromal cells obtained from patients with FOP exhibit increased mTOR signalling [100, 102]. Furthermore, the mTORC1 signalling pathway was activated by activin A via ectonucleotide pyrophosphatase/phosphodiesterase 2 (ENPP2), which is a recognized mTOR signalling activator. In addition, rapamycin-induced mTOR inhibition inhibited chondrogenesis in vitro and HO in FOP model mice that were induced with activin A [100, 102]. These findings demonstrate the critical role of the ACVR1 mutation-induced ENPP2/mTOR signalling axis, which contributes to FOP formation [100, 102]. Interestingly, as previously described, mTORC1 signalling can positively regulate the downstream NF-κB pathway to promote chondrogenesis mediated by MSCs [62]. Therefore, mTORC1-induced NF-κB signalling activation can induce the expression of NF-κB-dependent chondrogenic genes, which can exacerbate FOP progression. During the chondrogenesis stage, FOP pathogenesis involves Activin-induced mTOR signalling, and NF-κB may be involved in chondrogenic regulation and aberrant BMP/Smad signalling regulation [100, 102].

## Therapeutics for treating HO associated with NF-κB signalling

HO is a gradual process in which inflammation is an important initiator, and consequent chondrogenesis promotes HO progression [129]. The cornerstone of modern therapy for HO is the use of anti-inflammatory medications such as steroids and nonsteroidal anti-inflammatory medicines (NSAIDs) [130–134]. These approaches try to reduce inflammatory responses and bone growth; however, they are not highly effective at preventing HO [16]. Therefore, new strategies to inhibit the inflammatory response and further ectopic bone formation are considered ideal and promising ways to treat or prevent HO. Since NF-κB is an essential signalling pathway for accelerating HO via these two processes, therapeutics targeting NF-κB might be promising for preventing HO progression [8].

### Drugs targeting inflammation-associated macrophages and their interaction with MSCs

#### tHO treatment

##### Metformin

Metformin, which is a synthetic dimethyl biguanide, has been commonly used as a first-line therapy for type II diabetes mellitus and was approved by the Food and Drug Administration (FDA). Interestingly, it has also been proven to attenuate HO progression predominantly during the inflammatory phase [8]. Studies have shown that metformin induces the polarization of macrophages to the anti-inflammatory M2 phenotype by activating AMPK signalling, thereby decreasing the proinflammatory M1 phenotype [8]. Research has shown that the phosphorylation of NF-κb p65 and IKBα at Ser32/36 is significantly increased at injury sites after HO induction, and AMPK activity decreases during HO progression to stimulate inflammation. Consistent with these findings, in macrophages isolated from FOP patients, increased NF-κB pathway activation was detected, and pharmacological suppression of canonical NF-κB signalling was abolished in an HO rat burn/tenotomy model [16, 33]. An in vitro study of the inhibitory effects of metformin on monocyte-to-macrophage transition showed that the expression of IL-10, TGF-β1 and BMP6 was significantly reduced when macrophages differentiated into the M2 phenotype and were treated with metformin. The underlying mechanism was assessed by western blot analysis and immunohistochemical staining, and the results revealed that metformin induced AMPK activation and decreased phospho-NF-κB p65 and phospho-IKBα levels, which reduced macrophage infiltration [8]. Overall, AMPK signalling was activated, and blockade of NF-κB signalling in M1/M2 macrophages was observed, as indicated by reductions in the levels of macrophage-secreted cytokines or mediators, including TGF-β1, BMP6, IL-10, TNF-α, MCP-1 and IL-6 [8]. Similarly, an in vivo study revealed that metformin could reduce the infiltration of M1 and M2 macrophages at injury sites. Metformin-treated HO mice exhibited decreased phosphorylation of NF-κB p65 and IKBαSer32/36 at injury sites, and AMPK activity was increased in injury sites [8].

**Fig. 7:**
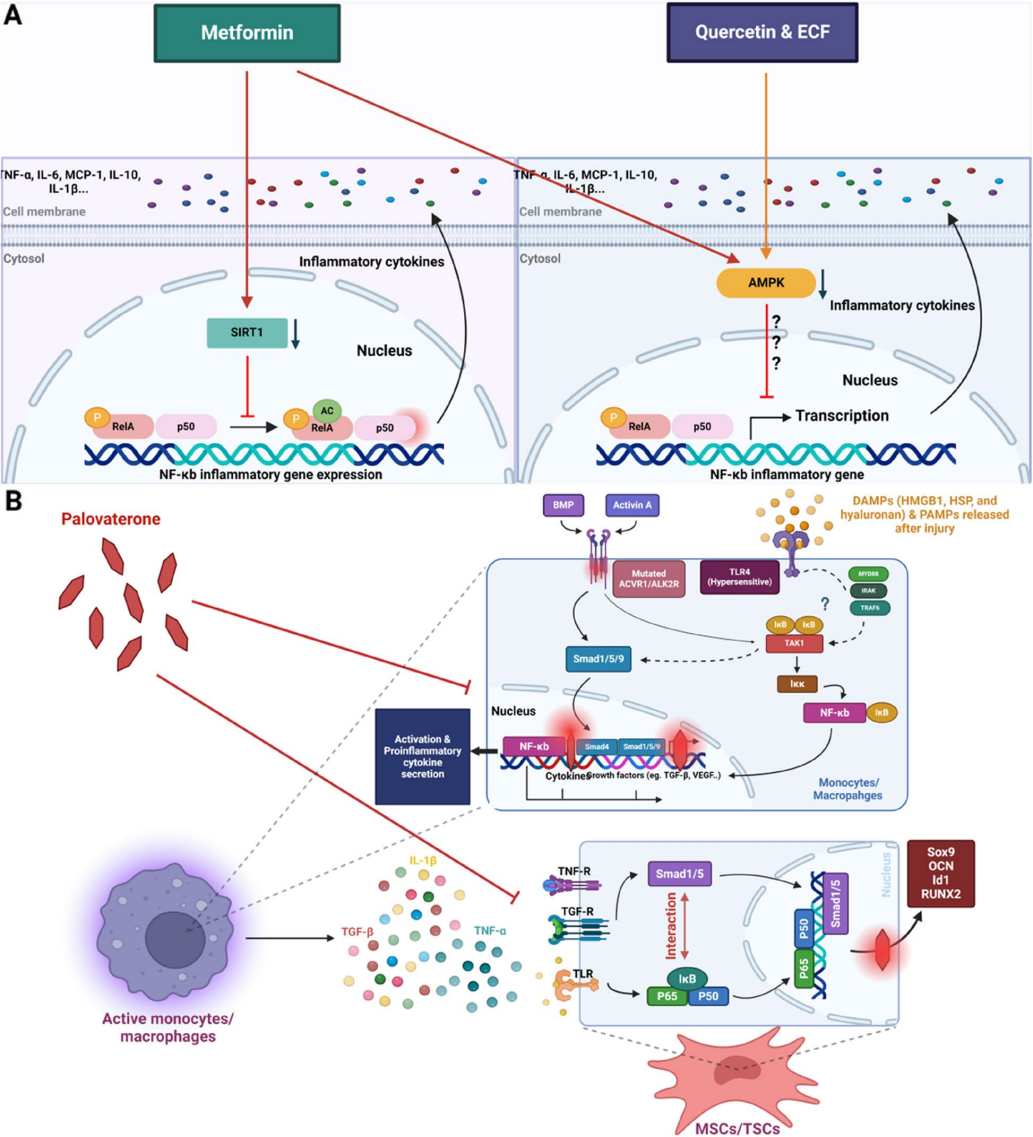
Schematic illustration of therapeutics of endochondral ossification and FOP targeting NF-κB signalling. **A** Endochondral ossification: therapeutics based on inflammatory responses. Metformin could both activate the SIRT1 and AMPK pathways to inhibit NF-κB-P65 activity to block inflammatory cytokine secretion, therefore repressing their capacity for infiltration and activation and other activities. Quercetin and ECF can activate AMPK to inhibit the transcriptional actions of NF-κB-dependent inflammatory genes. **B** FOP: therapy based on inflammatory responses and MSC/TSC differentiation. During inflammation, the ACVR1 R206H mutation exuberantly stimulates smad1/5/9 phosphorylation. Furthermore, smad4 is recruited to form a complex with smad1/5/9, which can interact with NF-κB signalling. FOP patients were found to show hyperreactivity of TLR4, which may also spark TAK1 and further NF-κB translocation to evoke synergistic roles with smad signalling. Interestingly, ACVR1-mediated signalling is also able to induce TAK1 activation. As a RARγ agonist, palovarotene interferes with smad complex formation to block inflammatory responses and MSC differentiation. Furthermore, in MSCs/TSC (tendon stem cell), it seems that palovarotene may also block the downstream transcriptional target of both NF-κB and smad, including OCN, Sox9, Runx2 and Id1, induced by macrophage-secreted molecules, including TGF-β and IL-1β, to further alleviate FOP (Created with BioRender.com)

Consistent with the anti-inflammatory effects of metformin, Sun’s study revealed that metformin could inhibit HO in a SIRT1-dependent manner to inhibit downstream NF-κB signalling, thereby suppressing inflammation progression [129]. An in vivo study showed that metformin could reduce HO 10 weeks after tendon injury. More importantly, in vivo and in vitro studies revealed that metformin could attenuate macrophage accumulation and inflammatory responses. Immunohistochemical staining revealed reduced macrophage infiltration, and the qRT‒PCR and enzyme linked immunosorbent assay (ELISA) results revealed reduced secretion of TNF-α, MCP-1 and IL-1β in the media after metformin treatment [129]. Furthermore, an in vivo study of the molecular mechanism revealed increased SIRT1 and deacetylated NF-κB p65 activity in injury sites [129]. SIRT1 is commonly known to exert anti-inflammatory effects by deacetylating downstream transcriptional regulators, including NF-κB [129]. Inhibition of SIRT1 in vitro and in vivo could also abrogate the inhibitory effects of metformin on HO, and treatment with the SIRT1 antagonist EX527 plus metformin could dramatically offset the inhibitory effects of metformin on inflammatory responses, including the secretion of cytokines, such as TNF-α and IL-1β, as well as macrophage infiltration in the early stage of HO [129]. An in vivo study showed that SRT1720 treatment could induce hypoactive chondrogenesis during HO, and treatment with EX527 plus metformin could facilitate chondrogenesis during HO. Immunofluorescence staining revealed decreased acetylated NF-κB p65 expression in the SRT1720-treated group and the metformin-treated group in the early stages of HO. This effect was dramatically prevented in the group that was administered EX527 plus the treatment, and the results showed the reversal of inflammatory responses, suggesting abrogation of the anti-inflammatory effects of metformin. These findings further confirmed that the effect of metformin was at least partially mediated by the SIRT1/p65 axis [129]. Therefore, the activation of SIRT1 by metformin inhibits NF-κB p65 activity to alleviate inflammation and the production of associated cytokines, including IL-1β. Additionally, research revealed a dose-dependent effect of metformin [129]. In conclusion, metformin can activate SIRT1 and AMPK signalling to prevent infiltration, polarization, activation and the secretion of inflammatory cytokines [8, 129]. In addition, since metformin can inhibit NF-κB signalling in macrophages, BMP signalling in MSCs may also be affected by crosstalk with macrophages via BMP6 and TGF-β to alleviate HO [8]. However, additional studies on human HO patients are needed to evaluate the efficacy and dose-related safety of these treatments and the associated side effects (Fig. 7).

##### ECF (ethyl caffeate) and quercetin

ECF, which is an essential compound found in Petunia, and quercetin, which is a natural polyphenol found in various fruits and vegetables, have been shown to exert anti-inflammatory effects by targeting the SIRT1/NF-κB axis [23, 24]. SIRT1 can directly deacetylate RelA/p65 at lysine 310 to modulate inflammatory responses [135]. Studies have shown that ECF and quercetin have strong binding affinities with SIRT1, and their binding can activate SIRT1 to inhibit NF-κB signalling; evidence suggest that inflammatory cytokines, including IL-6 and TNF-α, are downregulated after treatment with ECF or quercetin, and SIRT1 activity is upregulated [23, 24]. Similar to metformin, the inhibitory effects of ECF and quercetin are dose-dependent [23, 24]. In summary, ECF and quercetin can inhibit the activity of macrophages by enhancing SIRT1 signalling to partially silence NF-κB signalling and specifically inhibit inflammatory factors by inhibiting polarization, infiltration and inflammatory cytokine secretion [23, 24].

In vivo, during murine HO model construction, SIRT1 expression was dramatically reduced. The inhibitory effects of quercetin on SIRT1 activation were examined, and the results revealed a decrease in macrophage-surface markers, including complement component receptor 3 alpha (CD11b) and cluster of differentiation 14 (CD14), as well as the depletion of inflammatory cytokines, such as MCP-1, IL-6, TNF-α, and IL-1β, during transition [23]. Additionally, quercetin inhibited the polarization of the M1 and M2 subtypes, which were substantially increased in response to burn/tenotomy stimuli. Similar to the cytokines involved in the transition process, inflammatory cytokines, including IL-10, MCP-1, IL-6 and other cytokines, were depleted, indicating that quercetin abolished inflammatory responses. Mechanistically, acetylated NF-κB p65 was significantly upregulated in response to traumatic stimuli and downregulated after quercetin treatment. Interestingly, this increase in the inflammatory environment could further activate NF-κB signalling in macrophages to form an inflammation-driven cycle leading to HO progression [64]. The relationship between SIRT1 and NF-κB was discussed in a previous section, and SIRT1 was the upstream molecule that modulated NF-κB activity. A study also revealed that SIRT1/NF-κB signalling occurred in mast cells in response to burn/tenotomy and could coordinate with macrophages to contribute to inflammatory responses. However, quercetin could dramatically inhibit mast cell activity via the NF-κB pathway axis [23] (Table 1).

**Table 1:** Potential therapeutics for endochondral ossification, including inhibitors and drugs targeting NF-κB-related signalling pathways

Inhibitors	Target	Target tissue	Main findings	Reference
Metformin	AMPK/SIRT1	Macrophages of HO rat	Activate AMPK or SIRT1 to block NF-κB activity, thus decreasing the secretion of inflammatory cytokines, preventing the infiltration, activation, and polarization of M1 macrophages and promoting M2 polarization.	[8, 129]
Quercetin	SIRT1	Macrophages and mast cells of HO mice	Activate SIRT1 to downregulate acetylated NF-κb p65, thereby inhibiting the transition from monocyte to macrophages and mast cells, the polarization of M1 and M2 subtypes and mast cell activity.	[23]
ECF	SIRT1	Macrophages of HO mice	Activate SIRT1 to downregulate acetylated NF-κb p65, thereby inhibiting macrophage polarization, infiltration and inflammatory cytokine secretion.	[24]
Palovarotene (FDA approved)	Smad pathway	Macrophages and MSC/TSCs of FOP rats/Humans	Decrease Id1, smad5, Sox9, Runx2, OCN and p65 expression; inhibit the binding of Smad1/5/8 and Smad4 to suppress the overactivation of NF-κb, eventually suppressing osteoblast differentiation and macrophage accumulation.	[127]
Pyrrolidinedithiocarbamate (PDTC)	NOS (Nitrous Oxide System)	Tendons of HO mice	PDTC could significantly inhibit the expression of p56 and inhibit the activity of the NF-kB pathway, significantly decreasing ectopic bone formation.	[33]
Rapamycin	mTORC1	Tendon cells of HO mice	Inhibit mTORC1, attenuate the progression of HO during the inflammation and early stage of chondrogenesis. Suppress early chondrogenic differentiation but promote hypertrophy and maturation at the later stage.	[62]
BAY11-7082	NF-κB p65	Tendon cells of HO mice	Inhibit NF-κB, reverse the chondrogenic differentiation of TDs due to the overactivation of mTORC1	[62]
JSH23	NF-κB p65	TDSCs	Inhibit nuclear translocation of NF-κB p65, rejuvenating senescent TDSCs induced by coculture with pyroptotic macrophages and slowing osteogenic induction	[25]
SRT1720	SIRT1	Macrophages and mast cells of HO mice	Activate SIRT1, decreasing the infiltration of monocyte-derived macrophages and mast cells during the early stages of HO	[23]

Quercetin might be a promising potential pharmacological agent for treating HO patients, since it can attenuate HO progression at an early stage. However, the effectiveness of quercetin was only assessed in a murine burn/tenotomy model established by classical methods. Unfortunately, in real clinical settings, bone injury and mechanical overload are rare, and it is difficult to determine clinical translation and interpretation prospects based on the effect of quercetin on murine models. To overcome these limitations and ascertain the pharmacological efficacy and safety of quercetin in humans, larger animals with human-like anatomy should be investigated [23].

In a mouse model of HO, ECF inhibited macrophage polarization, infiltration and inflammatory cytokine secretion via the SIRT1/NF-κB signalling pathway in vitro and in vivo, similar to quercetin [24]. An in vivo study revealed that ECF could also block the expression of osteogenic genes, including ALP, Runx2 and OCN [24]. Additionally, ECF was shown to inhibit ectopic bone formation by preventing inflammatory media formation, rather than by directly inhibiting the osteogenic differentiation and mineralization of MSCs [24]. A study also showed strong binding energy between ECF and SIRT1, and ECF could directly activate SIRT1. Interestingly, unlike metformin and quercetin, which act in a dose-dependent manner, ECF not only inhibits p65 acetylation in a dose-dependent manner but also in a time-dependent manner, and the effect occurs within minutes [24].

Similar to quercetin, the effects of ECF on HO were examined only in a mouse model of HO, and the pharmacological effects of ECF on humans still require trials and safety checks.

#### FOP treatment

##### Palovarotene

Palovarotene is a novel HO inhibitor that can be used to treat FOP. Osteogenic progenitor cells, osteogenic signal transduction pathways, and the local tissue microenvironment are key factors in current research on HO pathogenesis [127]. The mechanism by which palovarotene inhibits HO could also involve these 3 factors. For example, TSCs function as osteogenic progenitor cells in an inflammatory microenvironment dominated by polarized macrophages; in addition, TSCs undergo osteogenic differentiation, during which the levels of bone deposition and phosphatase are increased, the NF-κB and smad pathways are activated, and NF-κB is transported into the nucleus where it promotes the transcription of NF-κB-dependent genes [127]. The transcription factors of NF-κB, including Smad1/5/8, are phosphorylated by ACVR1 after activin binding, and phosphorylated Smad1/5/8 binds with Smad4 and enters the nucleus to regulate gene expression [128]. Ultimately, HO can be induced. After palovarotene treatment, the levels of many molecules, such as Id1, smad5 and p65, are significantly decreased in HO animal models with inflammatory symptoms. Id1 and smad5, which are key molecules in the smad pathway, represent the activity of this signalling pathway [136, 137]. Palovarotene can bind to retinoic acid receptor (RAR) agonists and inhibit the interaction between Smad1/5/8 and Smad4 to suppress the overactivation of NF-κB, which occurs during the pathogenesis of FOP. Additionally, a study revealed that palovarotene could inhibit downstream target genes of Smad and NF-κB to eventually suppress osteoblast differentiation and macrophage accumulation [127].

Recently, palovarotene has been approved for sale by the FDA. A study assessed the efficacy and safety of palovarotene in patients with FOP. The probability of any reduction in new HO cases treated with palovarotene was 99.4% in one analysis. However, safety concerns were observed during palovarotene treatment. The patients reported at least one adverse event, and 97% reported retinoid-associated adverse events. Additionally, 29.3% of patients under 14 years of age experienced serious adverse events, including premature physeal closure (PPC) or epiphyseal disorder. Post hoc computational analyses using whole-body computed tomography (WBCT) revealed decreased vertebral bone mineral density, content, and strength, as well as an increased risk of vertebral fractures in palovarotene-treated patients [138]. Further studies are needed to better understand the risks and benefits of palovarotene treatment of FOP patients [138].

### Therapeutics that control osteoclast differentiation in monocytic precursors

A study showed that NF-κB signalling could exert antiapoptotic effects on osteoclasts via BMP signalling [139]. The research team designed specific monocytic precursors, and under the control of CID and AP20187, these cells differentiated into osteoclasts. Activation of NF-κB signalling in osteoclasts in local injury sites in soft tissue can help prevent ectopic ossification induced by traumatic stimuli, absorb calcified substances and therefore dissolve ectopic bones [140].

## Discussion and perspectives

HO has received increasing attention in clinical settings, and HO patients have an increased tendency to have unfavourable prognoses, as indicated by restricted joint movement and constant pain caused by neural nerve compression by ectopic bones [1]. Therefore, understanding or discovering additional clues about the molecular mechanism of HO can provide additional opportunities for developing HO therapeutics. In this review, we focused on the roles of the NF-κB signalling pathway in endochondral ossification (a type of HO), and split the process into 3 stages: the initial inflammation mediated by macrophages, subsequent crosstalk between macrophages and MSCs, and MSC-mediated chondrogenesis and osteogenesis during endochondral ossification [8, 25, 141].

During the first phase, canonical NF-κB signalling is an essential proinflammatory pathway that induces activation, polarization, infiltration, and inflammatory cytokine secretion in macrophages to establish an inflammatory microenvironment for further osteogenic events [8, 23]. During this process, SIRT1 in macrophages acts as an upstream negative regulator, and a decrease in SIRT1 expression leads to increased macrophage activation, polarization, infiltration and cytokine secretion [23]. Additionally, NLRP3 inflammasome activation was also observed in pyroptotic macrophages, which can regulate the NF-κB signalling pathway and mediate the secretion of IL-1β and HMGB1, which can further activate TDSC senescence and differentiation [25]. NF-κB can also interact with other signalling pathways, such as the AMPK pathway, and a reduction in AMPK during HO could disinhibit NF-κB signalling, thereby promoting macrophage activation, infiltration and cytokine secretion. These findings demonstrate the effects of NF-κB signalling on inflammatory responses [8]. In the interaction stage, which is mediated by macrophages and MSCs, inflammatory cytokines, including TNF-α, IL-1, IL-6, MCP-1 and IL-1β, which are controlled by NF-κB and expressed in macrophages, further stimulate MSC differentiation via the activation of BMP signalling and NF-κB signalling [8, 25]. During the chondrogenesis stage, NF-κB signalling can modulate the expression and activity of Sox9, which targets the promoter region of NF-κB-p65, as well as RSPO2, and RSPO2 expression can inhibit HO progression [64, 84]. Moreover, PI3K/AKT signalling induced by GH and IGF-1 and mTORC1 signalling induced by stretch stimuli can regulate NF-κB signalling in chondrocytes to promote chondrogenesis [62, 66–70]. During the osteogenesis stage, NF-κB signalling was shown to inhibit osteoblast development during osteogenesis [1, 97]. The control of NF-κB signalling has been examined, and mTOR signalling can favourably trigger NF-κB signalling to modify osteogenesis in response to mechanical stimuli [97]. Furthermore, osteoblast differentiation can be inhibited by integrin-mediated NF-κB signalling activation independent of the mTOR-NF-κB axis [99]. Furthermore, BMP signalling was shown to cooperate with mTOR signalling and inhibit NF-κB signalling to regulate osteogenesis [100–102]. However, the SPARC protein can regulate NF-κB signalling, modulating classical NF-κB signalling in osteoblasts to promote osteoblast differentiation [97, 103]. These findings suggest that different regulatory effects occur via different modulators that can affect NF-κB activity to exert different effects. In addition, in FOP, ACVR1 mutation-induced high TLR4 sensitivity can activate NF-κB signalling and interact with smad signalling in macrophages to promote inflammation in MSCs, and mTOR signalling may be involved in chondrogenic and osteogenic commitment to promote ectopic bone formation during FOP [16, 100, 102, 126, 128].

Because NF-κB signalling plays an important role in HO, there have been pharmacological studies targeting NF-κB signalling. In traumatic HO, metformin, quercetin and ECF indirectly activate SIRT1 or AMPK signalling to further inhibit NF-κB signalling in macrophages [8, 23, 24]. However, most of the therapeutic effects have been evaluated at the animal level, but safety and side effect have not been assessed [8, 23, 24]. Further research is needed to identify likely candidate drugs for clinical trials to consider potential side effects. For FOP, palovarotene can inhibit the interaction between BMP signalling and NF-κB signalling [128]. A published study showed that Palovarotene passed the phase III MOVE clinical trial conducted on FOP patients and was approved by the FDA; this trial showed the significant ability of palovarotene to attenuate FOP progression, which is promising for further applications in the clinic [138]. Therefore, in the future, additional research should be performed to better assess the clinical viability of these drugs, which are expected to improve the prognosis of HO patients by providing new therapeutic options to block HO progression in the early inflammation phase.

However, there are still gaps in the knowledge of NF-κB signalling in HO. For example, during chondrogenesis and crosstalk between macrophages and MSCs, some studies have focused on NF-κB signalling in TDSCs, which are tendon/ligament-derived stem cells and specific types of soft tissue-originating cells [25, 127]. This may not be consistent with common MSCs. The roles of NF-κB signalling in osteoblasts in the context of HO are still unclear. For instance, a number of studies have demonstrated that the activation of canonical NF-κB signalling prevents bone formation by inhibiting osteoblast differentiation caused by an increase in TNF, and endogenous TNF-α lowers the maximum peak of bone mass and inhibits osteoblastic Smad activation through NF-kappaB [116, 142–144]. The molecular mechanism may involve the suppression of special AT-rich sequence-binding protein 2 (SATB2), which can be induced by BMP-2 signalling to further block osteogenic gene expression [98]. In the context of HO, TNF-α, which is a classical cytokine, is an important component of the inflammatory environment and is crucial for further chondrogenesis and osteogenesis [25]. This indicates the complex mechanism of TNF-α in different stages and environments. Moreover, according to a recent study, bone mass temporarily increases in young mice when canonical signalling in mature osteoblasts is inhibited by genetic modification [117]. Further proof that canonical NF-κB signalling negatively affects bone formation showed that an NF-κB inhibitor increased the healing of murine calvarial defects and improved the density of bone minerals in ovariectomized animals [145]. In contrast, multiple studies have shown that BMP-2-mediated upregulation of Osterix (Osx) and Runx2 increased the differentiation of mesenchymal progenitor cells (MPCs) into osteoblasts through TNF-induced activation of canonical NF-κB signalling [146–148]. These findings suggest that complicated interactions between cytokines and canonical NF-κB signalling can have negative or positive regulatory effects on osteoblasts to alter bone mass, and these effects depend on the type of stimulus and stage of osteoblastic cell development [142]. However, these findings were not specifically conducted under HO conditions. During the early stage of chondrogenesis in the ectopic bone formation stage of HO, NF-κB signalling activation is important for driving MSC differentiation and proliferation [19, 25]. Subsequent osteoblast differentiation was likely driven by the inhibition of NF-κB signalling [142]. Therefore, further research on the osteoblast differentiation stage should be performed under HO conditions to uncover additional details about the roles of NF-κB signalling. In addition to canonical NF-κB signalling, alternative NF-κB signalling pathways may play roles in osteoblasts [142]. RelB plays a crucial role in preventing osteoblast differentiation, and ablation of p100 and RelB prevents osteopenia in p100-null mice and even increases bone mass and osteoblast surfaces [142, 149]. However, this topic has not been examined in the HO background; therefore, research on noncanonical NF-κB signalling during HO might also provide additional clues to the molecular mechanism of osteogenesis during HO. Finally, current studies on therapeutics targeting NF-κB signalling have focused on inflammatory stages [8, 23, 24, 127]. Therefore, future pharmacological studies on the early osteogenic stage (chondrogenesis) might be useful for blocking HO progression. However, osteogenesis is a gradual process in which final ectopic bone maturation can only be treated with surgical excision to partially restore joint motility; therefore, the early chondrogenesis stage might be the optimal curative window [150, 151]. Therapeutic studies on NF-κB signalling during chondrogenesis might be helpful. Therefore, additional research is urgently needed to decipher the process and mechanism of HO and shed light on the development of novel therapies.

## Summary

Heterotopic ossification (HO) is a pathological process in which ectopic bone develops in soft tissues in the skeletal system. NF-κB signalling is essential for HO and drives initial inflammation through interactions with the NLRP3 inflammasome, SIRT1, and AMPK. NF-κB signalling also promotes chondrogenesis through interactions with mTOR, PI3K/AKT, RSPO2, and SOX9. NF-κB expression can influence osteoblast differentiation through SPARC regulation, mTOR/BMP signalling, or integrin-mediated signalling in response to stretch stimuli in the final osteogenic stage. During FOP, mutant ACVR1-induced NF-κB signalling can exacerbate inflammation in macrophages and promote chondrogenesis and osteogenesis in MSCs through interactions with smad signalling and mTOR signalling.

## Data Availability

No datasets were generated or analysed during the current study.

## Abbreviations

HO: Heterotopic ossification
tHO: Traumatic HO
FOP: Fibrodysplasia ossificans progressive
NF-κb: Nuclear transcription factor kappa B
NLRP3: NOD‐like receptor protein 3
SIRT1: Sirtuin1 protein
AMPK: AMP-activated protein kinase
mTOR: Mechanistic target of rapamycin
PI3K/AKT: Phosphatidylinositol-3-kinase/Protein kinase B
SPARC: Secreted protein acidic and rich in cysteine
MSC: Mesenchymal stem cells
ACVR1: Activin A receptor type I
GS: Glycine-serine
BMP: Bone morphogenetic protein
THA: Total hip arthroplasty
HIF1-α: Hypoxia inducible factor 1-α
TGF-β: Transforming growth factor-β
RAR: Retinoic acid receptor
RHD: Rel homology domain
IκB: Inhibitor kappa B protein
TNF-α: Tumor necrosis factor-alpha
LPS: Lipopolysaccharides
IKK: IκB kinase
NIK: NF-κb-inducing kinase
MIP1-α: Macrophage inflammatory protein 1α
MCP-1: Monocyte chemoattractant protein-1
TLR4: Toll-like receptor 4
PGC-1: Peroxisome proliferator-activated receptor- γ coactivator
FOXO: Forkhead box O
JAK/STAT: Janus kinase/signal transducer and activator of transcription
MAPK: Mitogen-activated protein kinase
NEK7: NIMA-related kinase 7
GSDMD: Gasdermin D
MyD88: Myeloid differentiation factor 88
IRAK: Interleukin 1 receptor-associated kinases
TAK1: Transforming growth factor-β-activated kinase 1
MAP3K7: TGF-Beta Activated Kinase 1
TAB2: Binding Protein 1
Rspo2: R-spondin 2
Sox9: SRY-box 9
Col2a1: Collagen Type II Alpha 1 Chain
HMGB1: High mobility group protein 1
TDSCs: Tendon-derived stem cells
SASP: Senescence-associated secretory phenotype
DAMP: Damage-associated molecular pattern
EVs: Extracellular vesicles
ARS: Alizarin red S
TRAF6: TNF receptor–associated factor
ALP: Alkaline phosphatase
Runx2: Runx family transcription factor 2
FGF: Fibroblast growth factor
Ihh: Indian hedgehog
Gli2/3: Glioma-associated oncogene1 protein 2/3
PIP2: Phosphatidylinositol – 4,5 – bisphosphate
PIP3: Phosphatidylinositol (3,4,5)-trisphosphate
TSC1/2: Tuberous sclerosis complex1/2
PDK1: Pyruvate dehydrogenase kinase 1
Bcl-XL: B-cell lymphoma-extra large
IGF: Insulin-like growth factor
GH: Growth hormone
CAG-EGFP-Rspo2: Cre-dependent transgenic mice
OPLL: Ossification of the posterior longitudinal ligament
ACAN: Aggrecan
Rac1: Ras-related C3 botulinum toxin substrate 1
OCNO: Steocalcin
MAPK-APPK2: Mitogen-activated protein kinase 2
HSP27: Heat shock protein 27
Osx: Osterix
JNK: Jun N-terminal kinases
PAMPs: Pathogen-associated molecular patterns
Id1: DNA Binding 1
ENPP2: Ectonucleotide pyrophosphatase/phosphodiesterase 2
RANKL: Nuclear Factor- κ B Ligand
NSAIDs: Nonsteroidal anti-inflammatory medicines
CD11b: Complement component receptor 3 alpha
CD14: Cluster of differentiation 14
WBCT: Whole-body computed tomography
PPC: Premature physeal closure
SATB2: Special AT-rich sequence-binding protein 2
MPCs: Mesenchymal progenitor cells
FDA: Food and Drug Administration
